# Epigenetic mapping of the somatotropic axis in Nile tilapia reveals differential DNA hydroxymethylation marks associated with growth

**DOI:** 10.1016/j.ygeno.2021.06.037

**Published:** 2021-06-30

**Authors:** Ioannis Konstantinidis, Dafni Anastasiadi, Pål Sætrom, Artem V. Nedoluzhko, Robin Mjelle, Tomasz Podgorniak, Francesc Piferrer, Jorge M.O. Fernandes

**Affiliations:** a Faculty of Biosciences and Aquaculture, Nord University, Bodø, Norway; b The New Zealand Institute for Plant and Food Research, Nelson, New Zealand; c Institut de Ciències del Mar, Spanish National Research Council (CSIC), Barcelona, Spain; d Department of Clinical and Molecular Medicine, Norwegian University of Science and Technology, Trondheim, Norway; e Department of Computer Science, Norwegian University of Science and Technology, Trondheim, Norway; f Bioinformatics core facility-BioCore, Norwegian University of Science and Technology, Trondheim, Norway; g K.G. Jebsen Center for Genetic Epidemiology, Norwegian University of Science and Technology, Trondheim, Norway

**Keywords:** DNA hydroxymethylation, Epigenetics, Somatotropic axis, Growth, Teleosts

## Abstract

In vertebrates, the somatotropic axis comprising the pituitary gland, liver and muscle plays a major role in myogenesis. Its output in terms of muscle growth is highly affected by nutritional and environmental cues, and thus likely epigenetically regulated. Hydroxymethylation is emerging as a DNA modification that modulates gene expression but a holistic characterization of the hydroxymethylome of the somatotropic axis has not been investigated to date. Using reduced representation 5-hydroxymethylcytosine profiling we demonstrate tissue-specific localization of 5-hydroxymethylcytosines at single nucleotide resolution. Their abundance within gene bodies and promoters of several growth-related genes supports their pertinent role in gene regulation. We propose that cytosine hydroxymethylation may contribute to the phenotypic plasticity of growth through epigenetic regulation of the somatotropic axis.

## 1 Introduction

Somatic growth requires a well-tuned transcriptional synchronization of three main tissue types that compose the somatotropic axis: the pituitary gland, liver and skeletal muscle. From the production of growth hormone (GH) in the pituitary gland to the stimulation and regulation of hundreds of genes in the liver and muscle, the somatotropic axis plays a critical role across several biological pathways involved in somatic growth, carbohydrate and lipid metabolism, energy equilibrium, normal development and reproduction [1–3]. The insulinlike growth factors (IGF-I and -II), their receptors (IGFRS) and binding proteins (IGFBPS) that are present in substantial amounts in liver and fast muscle, are directly associated with GH and collectively regulate somatic growth. However, the interaction of these molecules is greatly affected by both endogenous and exogenous factors, i.e. sex, developmental stage, nutrition, genetic background, temperature, photoperiod, salinity, dissolved oxygen, immunocompetence and stress [4–7]. For example, previous research has shown that seasonal differences [8], photoperiod, and sexual maturity [9] affect insulin-like growth factor 1 (*igf1*) plasma levels and consequently somatic growth. In addition, the liver is the main source of *igf1* production; however, in cases of malnutrition the liver is predominantly unresponsive to GH stimulation [10]. Such interactions have been widely demonstrated and both endocrine and paracrine *igf1* have been linked to bone, cartilage and muscle growth across vertebrates [11].

Because growth rate varies considerably with environmental factors [12], epigenetic mechanisms, which are known to integrate genomic and environmental information to bring about the phenotype [13], have the potential to be major regulators of growth both in early development (e.g., thermal imprinting) [14–16], and adulthood. Indeed, intrauterine growth restriction in rats induces DNA methylation changes around growth hormone response elements of the IGF-1 gene, resulting in developmental re-programming and persisting long-term effects [17]. Additionally, epigenetic mechanisms are likely responsible for splicing events leading to the translation of tissue-specific IGF-1 peptides through the usage of alternative promoters and exons [18,19]. In teleosts, recent studies have shown that epigenetic modifications regulate the expression of important growth genes during temperature fluctuations such as the DNA methylation of *myogenin* in Atlantic salmon (*Salmo salar*) [20] and Senegalese sole (*Solea senegalensis*) [14,21]. We have recently reported that growth-related methylation differences in *igf2bp2* are likely linked to phenotypic differences between small and large Nile tilapia full-sibs [22]. We identified sex-specific DNA methylation marks within the *map3k5, akt3, gadd45g* and *ppargc1a* genes. Their epigenetic regulation could potentially explain size-related differences among sexes because of their involvement in processes such as cell cycle, proliferation, cellular response to glucose and muscle tissue development [22]. The epigenetic function of non-coding RNAs, such as microRNAs (miRNAs), has also been shown to play critical roles in somatic growth [15]. For example, miR-206 targets the 3’UTR of IGF-1 and directly affects its expression in Nile tilapia [23], while miR-1 and miR-133 regulate the sarcomeric actin organization in zebrafish [24]. Similar results have been shown in mammalian cells, such as hepatocellular carcinoma cells, where miR-190b directly regulates IGF-I expression [25]. DNA methylation plays also an important role within the GH/IGF-I axis. Recent studies have identified the P2 promoter of IGF-I to be epigenetically mediated by DNA methylation with significant consequences in IGF-I circulation and human growth [26,27]. Although DNA methylation is an epigenetic modification that has received much attention in response to several biological contexts, including the function and regulation of major components of the somatotropic axis, little is known for its oxidized derivative. DNA hydroxymethylation is an epigenetic modification that occurs via ten-eleven-translocation enzymes, which catalyse the oxidation of 5-methyl (-CH_3_) into 5-hydroxymethyl (-CH_2_OH) groups in cytosines (5hmCs) [28]. Additionally, 5hmC has been identified as a stable DNA modification with a pertinent role in gene regulation and transcription [29,30], tissue-specific patterns and developmental stage-dependent profiles [31]. In human, 5hmCs are not exclusively correlated with DNA methylcytosine (5mC) content and when found in transcribed gene bodies they have been positively correlated with transcription [32]. In spite of 5hmC’s significance in regulating gene expression in humans [31–33] and mice [34,35], little is known about its role on growth, especially taking into account that it has been associated with abnormal growth, cancers and oncogenic pathways such as the PI3K-Akt [36,37].

Growth hormone signaling and the overall function of the somatotropic axis is largely conserved through evolution between fishes and higher vertebrates [38]. In contrast to mammals, most teleosts are poikilothermic and thus their growth rate is influenced to a greater extent by variations in environmental factors such as temperature. Furthermore, mammals have a determinate growth in comparison to many teleost species, which exhibit indeterminate growth. Together, the functional conservation of the somatotropic axis and the peculiarities described above, make teleosts excellent models for the investigation of epigenetic mechanisms that modulate the somatotropic axis. In this study, we compared genome-wide and tissue-specific 5hmC profiles at single nucleotide resolution between pituitary, liver and muscle using Nile tilapia (*Oreochromis niloticus*) as our model organism. Finally, we investigated the link between 5hmC and gene expression in skeletal muscle, since it constitutes the majority of the body mass and is directly related to growth.

## 2 Results

### 2.1 Tissue-specific 5hmC levels across the somatotropic axis

Pituitary, liver and muscle samples were collected from five Nile tilapia male siblings that were reared for 8 months in common garden. Among the three main tissues that comprise the somatotropic axis, 5hmC levels were highest in liver, followed by muscle and pituitary. The overall 5hmC levels of each tissue followed the sequencing depth of the corresponding reduced representation 5hmC profiling (RRHP) libraries (see Methods). Pituitary libraries had an average depth of 21.7 M raw reads compared to 28.1 M and 22.6 M raw reads in liver and muscle libraries, respectively. After quality control, adapter trimming and alignment of the sequenced reads to the Nile tilapia genome, an average of 10.6, 14.9, and 11.5 M reads were obtained from the libraries prepared from pituitary, liver and muscle, respectively (Fig. S1).

**Fig. 1:**
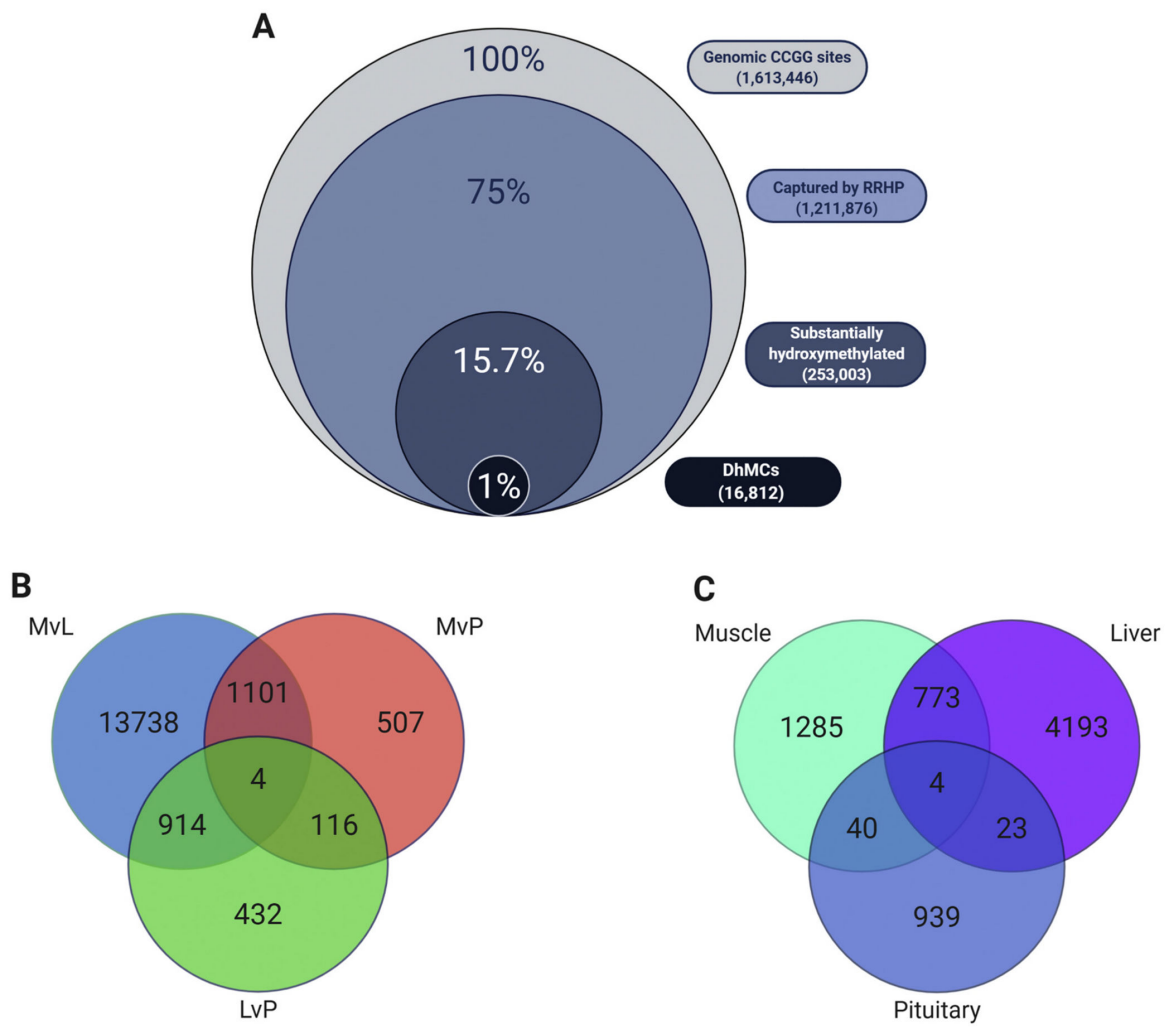
Venn diagrams representing the RRHP dataset. A) Stacked Venn diagram showing the distribution of total CCGG sites captured by RRHP, substantially hydroxymethylated and differentially hydroxymethylated among tissues compare to the total genomic CCGG sites of the Nile tilapia genome. B) Venn diagram depicting total differentially hydroxymethylated sites between and among tissue comparisons (MvL – Muscle vs Liver, MvP – Muscle vs Pituitary, and LvP – Liver vs Pituitary). C) Venn diagram showing the total and shared number of hyper-hydroxymethylated genes among tissues. Genes containing or associated with a DhmC in close proximity based on their distance to TSS were considered differentially hydroxymethylated.

The Nile tilapia genome contains 1,613,446 CCGG sites in both strands across its 22 linkage groups and the mitochondrial genome. RRHP captured 75% (1,211,876 CCGG) of all possible sites. The reduced coverage was mostly attributed to the size selection during library preparation. Compared to the total number of genomic CCGGs, 15.7% was significantly hydroxymethylated based on the median filtering of 5hmC counts (see Methods). The remaining 59.3% was dominated by 5hmC sites with very low counts across all tissues. A total of 16,812 5hmCs, which was approximately 1% of the total genomic CCGGs, were identified as differentially hydroxymethylated among the three tissues (Fig. 1A).

The highest difference was detected between the muscle and liver (MvL), with 15,757 cytosines being differentially hydroxymethylated, out of which 3119 were hyper-hydroxymethylated in muscle (q < 0.01). By comparing the hydroxymethylation profiles between muscle-pituitary (MvP) and liver-pituitary (LvP) we identified 1728 (q < 0.05) and 1467 (q < 0.01) additional DhmCs, respectively (Fig. 1B). An overlap of almost 2000 DhmCs was observed between pairs (MvL-LvP, MvL-MvP and LvP-MvP); however, we identified only four cytosines with significantly different hydroxymethylation levels among all pairwise comparisons (Table S1). Two of these four DhmCs were found within predicted long non-coding RNAs (*LOC102076435* and *LOC112847764*), one DhmC located within the predicted gene gap junction delta-3 protein-like (*LOC100690349*) and one DhmC within the par-3 family cell polarity regulator alpha, b (*pard3ab*).

### 2.2 Tissue-specific DNA hydroxymethylation abundance within genomic features

**Fig. 2:**
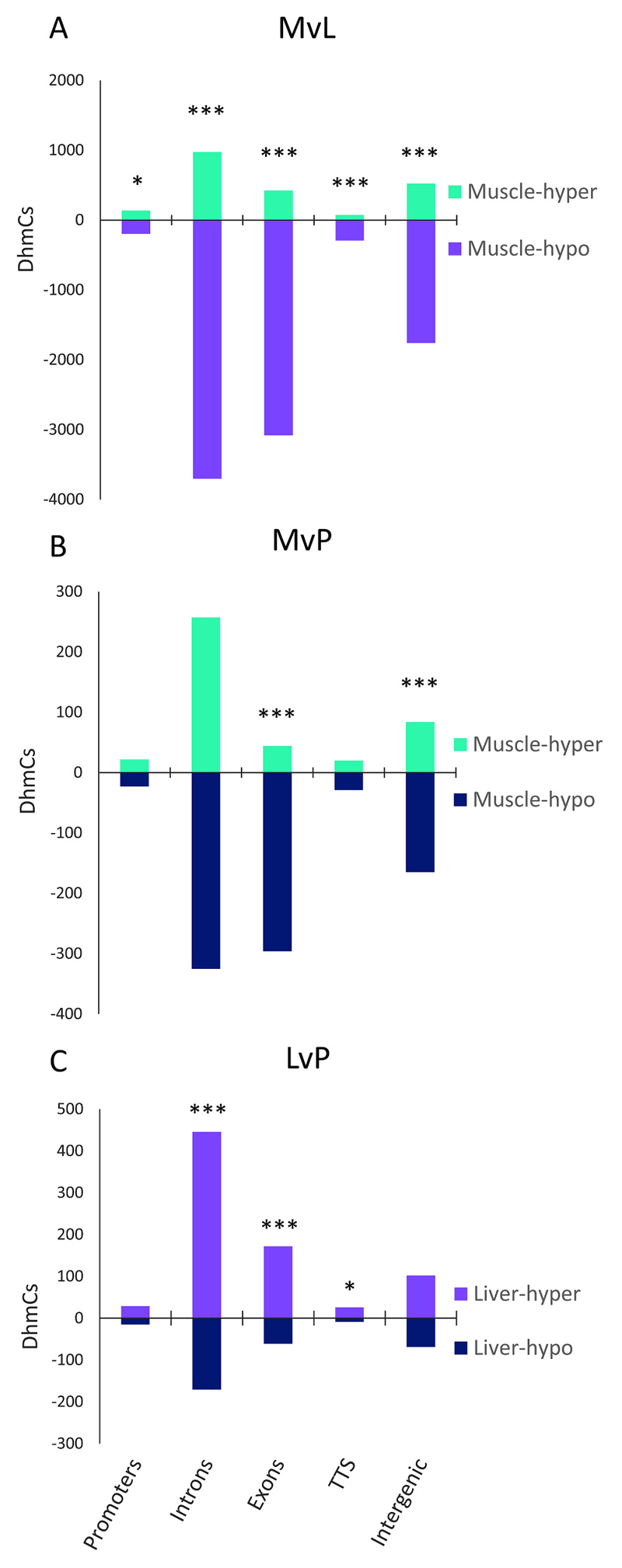
Histograms depicting the number of differentially hydroxymethylated cytosines (DhmCs) per pairwise comparison and annotated feature. Pairwise tissue comparisons are presented as (A) for muscle versus liver, (B) muscle versus pituitary, and (C) liver versus pituitary. The number of hyper- and hypo-hydroxymethylated sites are shown separately for promoters, introns, exons, transcription termination sites (TTS) and intergenic regions. *P*-values were calculated using Fisher’s test with 95% confidence interval and asterisks depict the *p*-value [* - (0.01 < *p* < 0.05); *** - (*p* < 0.001)].

Although DNA hydroxymethylation levels were not significantly different among tissues due to high variation among individuals, we identified significant differences in DhmC enrichment within the annotated features of the Nile tilapia genome. Compared to liver, the muscle was characterized by an overall hypo-hydroxymethylation across all the annotated features (*p* < 0.001), including promoter regions (*p* < 0.05; Fig. 2A). Striking differences were also observed between pituitary and muscle, with exons and intergenic regions being mostly hypo-hydroxymethylated in muscle (*p* < 0.001; Fig. 2B). By comparing liver to pituitary, we observed that the liver genome was hyper-hydroxymethylated across all the annotated features with significant enrichment within introns and exons (p < 0.001) and transcription termination sites (*p* < 0.05; Fig. 2C). The relative enrichment of DhmCs was calculated based on the number of 5hmCs with the highest 5hmC levels in each annotated feature per tissue. Compared to liver and pituitary, DhmCs with the highest levels of hydroxymethylation in muscle were relatively enriched in introns (12%) and promoters (104%), whereas exons were depleted by 58%.

In contrast, DhmCs with highest levels of hydroxymethylation in liver were relatively depleted within promoters and enriched within exons, compared to muscle and pituitary (Fig. S2). The highest percentage of DhmCs among all tissues was detected within introns (40% on average), while DhmCs in intragenic regions including promoters represented 70%, 74.6% and 71.8% of the entire dataset in muscle, liver, and pituitary, respectively.

### 2.3 Differentially hydroxymethylated cytosines are found in genes with distinct molecular functions in each tissue

To better understand whether DhmCs were linked to tissue-specific genes and potentially their functions, we performed a gene ontology enrichment analysis based on the genes containing or being closely associated to DhmCs. In total, we identified 2102 hyper-hydroxymethylated genes in muscle compared to liver and pituitary, 4993 genes in liver compared to muscle and pituitary and 1006 genes in the pituitary compared to muscle and liver. Interestingly, we identified 773 genes containing hyper-hydroxymethylcytosines in both the muscle and liver, 40 common genes between muscle and pituitary and 23 genes shared between liver and pituitary (Fig. 1C).

In muscle compared to liver, genes containing hypo-5hmCs (lower 5hmC levels in muscle) were associated with molecular functions related to transcription regulator and GTPase activator activity, GTPase binding and Ras GTPase binding. Gene ontologies related to development as well as anatomical structure and system development were among the most enriched biological processes. Furthermore, we identified 12 biological processes linked to metabolism, including regulation of primary, cellular, macromolecule, RNA and nitrogen compound metabolic process. Interestingly, hypo-5hmCs were assigned to gene ontologies such as skeletal system, muscle structure and cartilage development (Table S2). On the other hand, hyper-5hmCs (higher 5hmC levels in the muscle) were primarily associated to ionotropic glutamate receptor activity, signaling receptor activity and cell surface receptor signaling pathway, as well as system development and developmental process (Table S3). Notably, 773 genes were identified containing both hyper- and hypo-5hmCs that occurred in different intragenic loci. These genes were primarily linked to the regulation of biological and cellular processes, as well as the signal transduction and response to stimulus (Table S4).

In muscle compared to pituitary, hyper-hydroxymethylated genes (higher 5hmC levels in the muscle) were associated with tissue morphogenesis and the morphogenesis of an epithelium, as well as the developmental process and signaling among other gene ontologies (Table S5). Hypo-hydroxymethylated genes (lower 5hmC levels in muscle) were enriched for functions related to transcription regulator activity and the regulation of metabolic and biosynthetic process of macromolecules and RNA (Table S6). We identified 40 shared genes between muscle and pituitary containing hyper- and hypo-5hmCs in different loci. However, due to the relatively low number of genes we did not identify any significant enrichment for molecular functions or biological processes.

Compared to pituitary, the liver contained hypo-hydroxymethylated genes (lower 5hmC levels in liver) that were enriched for a cell surface receptor signaling pathway and axon target recognition (Table S7). In contrast, hyper-hydroxymethylated genes (higher 5hmC levels in the liver) were enriched for molecular functions related to protein and DNA binding. The most enriched biological processes were the regulation of cellular and biological processes, signaling and signal transduction (Table S8). In total, 23 genes containing hyper- and hypo-5hmCs were found within the LvP pairwise comparison. Among them, we identified the gene coding for arginine-glutamic acid dipeptide repeats protein (rere), which is involved in cell survival and apoptosis [39], and the protein phosphatase 1 regulatory subunit 3C (*ppp1r3c*) involved in glycogen metabolism as well as the metabolic process of macromolecules [40].

### 2.4 Differentially hydroxymethylated genes involved in somatic growth

**Fig. 3:**
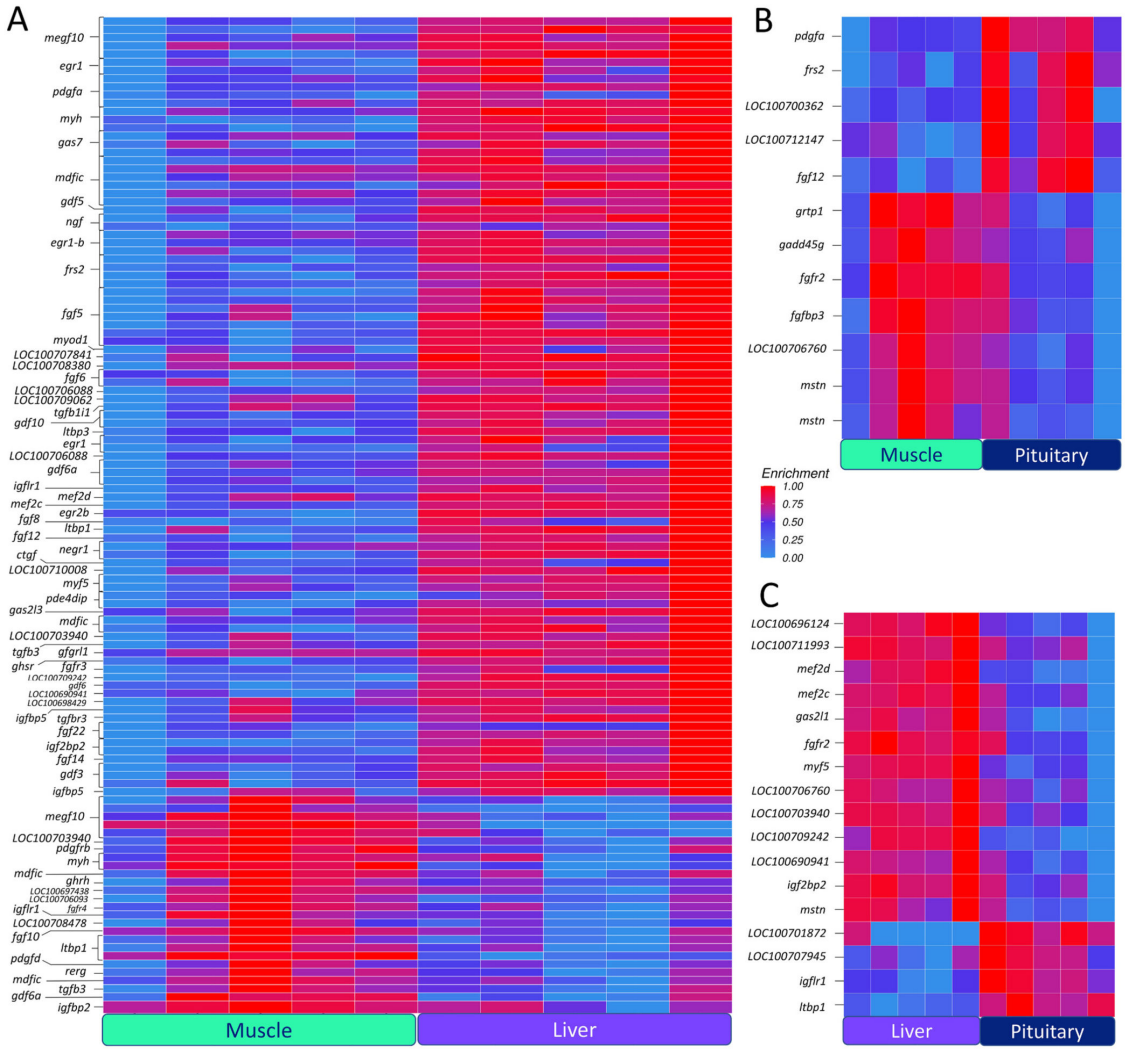
Heatmaps of growth-related genes containing differentially hydroxymethylated cytosines (DhmCs) among the three tissues. The level of 5hmC enrichment is mapped based on 5hmC counts across samples. The three heatmaps represent the comparisons between (A) muscle and liver, (B) muscle and pituitary, and (C) liver and pituitary. The 5hmC enrichment is represented as a gradient from light blue (low) to red (high). (For interpretation of the references to colour in this figure legend, the reader is referred to the web version of this article.)

Genes that play a critical role in development and somatic growth were identified across all pairwise comparisons. Most genes involved in somatic growth were hypo-hydroxymethylated in muscle compared to liver. These included several fibroblast growth factors (*fgf5*, *fgf6, fgf8, fgf12, fgf14* and *fgf22*), the receptor *fgfr3* and the fibroblast growth factor receptor substrate 2 (*frs2*). Only *fgf10* and *fgfr4* were hyper-hydroxymethylated in muscle. Additionally, growth arrest-specific proteins (*gas2l3* and *gas7*) and growth differentiation factors (*gdf3*, *gdf5, gdf6, gdf6a* and *gdf10*) were also hypo-hydroxymethylated in muscle. Hypo-5hmCs were detected within the last exon of growth hormone secretagogue receptor type 1 (*ghsr*), the second intron of insulin-like growth factor 2 mRNA binding protein 2 (*igf2bp2*), the first and third introns of insulin-like growth factor binding protein 5 (*igfbp5*) and the 32nd intron of IGF like family receptor 1 (*igflr1*). On the other hand, the promoter of the growth hormone releasing hormone (*ghrh*), the first intron of the insulin-like growth factor binding protein 2-B and the 29th exon of *igflr1* contained a single hyper-5hmC. Several genes involved in somatic growth contained both hyper- and hypo-5hmCs in different intragenic loci. These included the myoD family inhibitor domain-containing protein (mdfic), the multiple epidermal growth factor-like domains protein 10 (*megf10*) and the myosin heavy chain fast skeletal muscle (*myh*) (Fig. 3A; Table S9A). In muscle compared to pituitary, we identified two hyper-5hmCs located 7549 and 7367 bp, from the transcription start site (TSS) of the myostatin (*mstn*) gene, and a single hyper-5hmC at 6945 bp from the TSS of the growth arrest and DNA damage inducible gamma (*gadd45g*) gene. Similar to the comparison between muscle and liver, fibroblast growth factor genes *fgf5, fgf12* and *frs2* were hypo-hydroxymethylated in muscle compared to pituitary; however, hyper-5hmCs were detected within the *fgfr2* and *fgfbp3* genes. Additionally, two hyper-5hmCs were identified within the 5th exon and 7th intron of the gene coding for myoD family inhibitor domain-containing protein 2 (*mdfic2*) (Fig. 3B; Table S9B). Several of the above-mentioned genes were also differentially hydroxymethylated in liver compared to pituitary (Fig. 3C; Table S9C).

### 2.5 Gene expression profiling in Nile tilapia fast muscle

We investigated the correlation between 5hmC levels and gene expression in muscle, since it constitutes the majority of the total body mass in Nile tilapia and it closely reflects growth rate. The average sequencing depth of the RNA-Seq libraries was 26 M reads (Table S10). Approximately 1 M reads were excluded from the analysis after quality control (Q > 20) and trimming. On average, 2.8 M reads were mapped multiple times and 19.2 M reads were mapped uniquely to the reference Nile tilapia genome. In total, we identified 80,417 transcripts that were ranked based on their normalized counts. The gene with the highest expression in Nile tilapia fast muscle was the actin alpha skeletal muscle A (*LOC100534413*), which is involved in several biological processes including muscle contraction, myosin binding, skeletal muscle fibre development and thin filament assembly. Among the genes with the highest expression, we identified several fast skeletal muscle myosin light and heavy chains (*mylpf, LOC100712344, LOC100707599, LOC100698429*), creatine kinases muscle a and b (*ckma* and *ckmb*), ATPase sarcoplasmic/endoplasmic reticulum Ca2+ transporting 1 like (*atp2a1l*) and several isoforms of troponin T (*LOC100707421*) and tropomyosin 1 (*tpm1*) (Table S11).

### 2.6 Association of muscle-specific gene expression and DNA hydroxymethylation

**Fig. 4:**
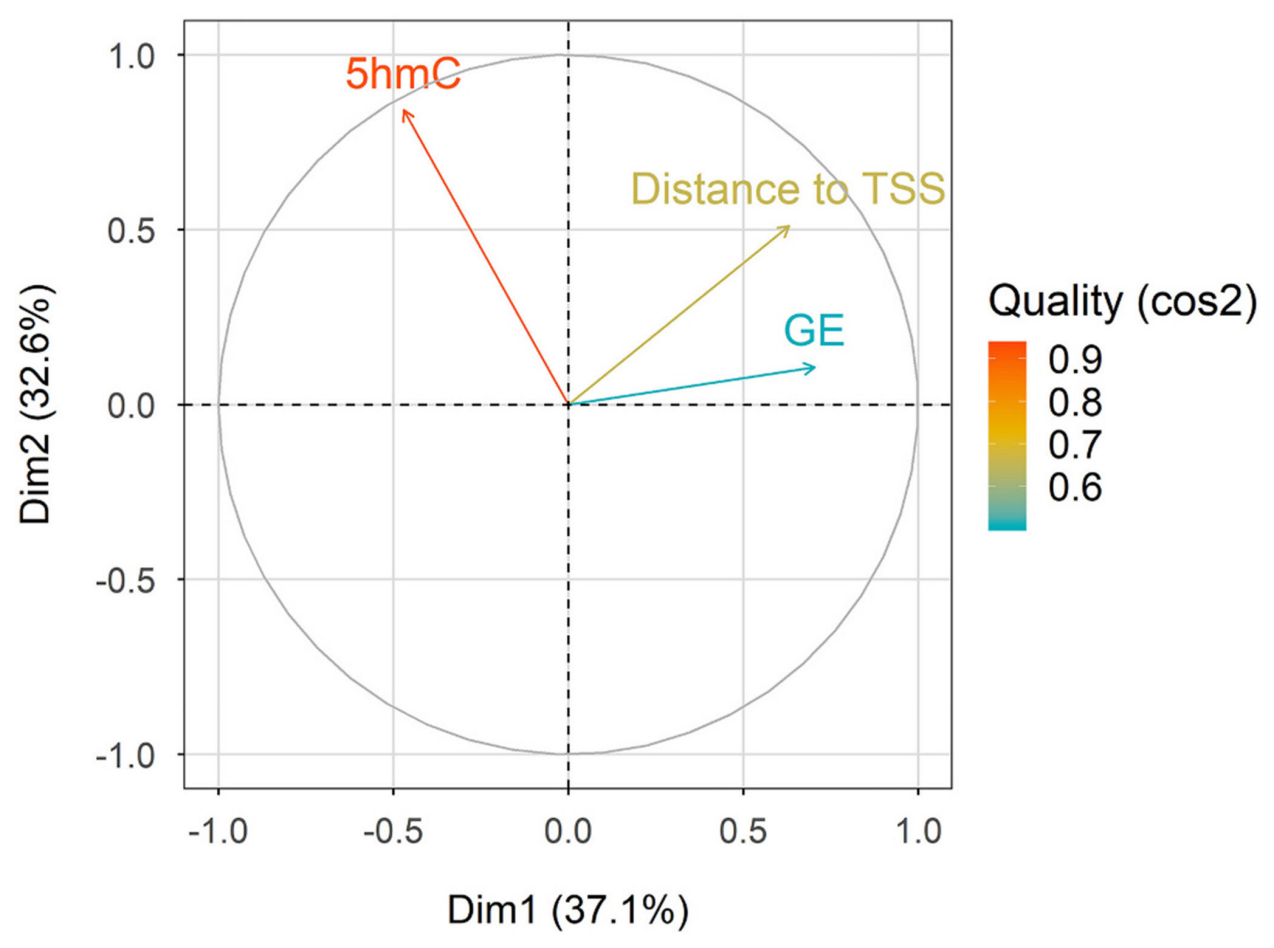
Principal component analysis using three variables. The three variables are the 5hmC level, gene expression (GE) corresponding to each 5hmC and their distance to the closest transcription start site, across the first two dimensions that explain 67.5% of the total dataset variation. Colour coding depicts the quality (cos^2^) of representation for the three variables, with lower, intermediate and higher quality represented in blue, yellow and orange, respectively. (For interpretation of the references to colour in this figure legend, the reader is referred to the web version of this article.)

To investigate the links between DNA hydroxymethylation and gene expression levels in muscle, we performed a principal component analysis using three variables: the 5hmC levels, the distance to the transcription start site (TSS) and gene expression. The first principal component (PC1) was strongly related to variation in both the distance to TSS (ρ = 0.63) and gene expression (GE; ρ = 0.70), indicating that these variables were strongly positively correlated in our data. On the other hand, 5hmC levels were strongly represented in the second principal component (PC2; ρ = 0.84) revealing no correlation with GE (ρ = 0.10; Fig. 4). Since the first kilobases up- and downstream of promoter regions have been previously correlated with gene transcription and alternative splicing [41–43], we also investigated the correlation between gene expression and 5hmC levels in promoters, first introns and first exons.

**Fig. 5:**
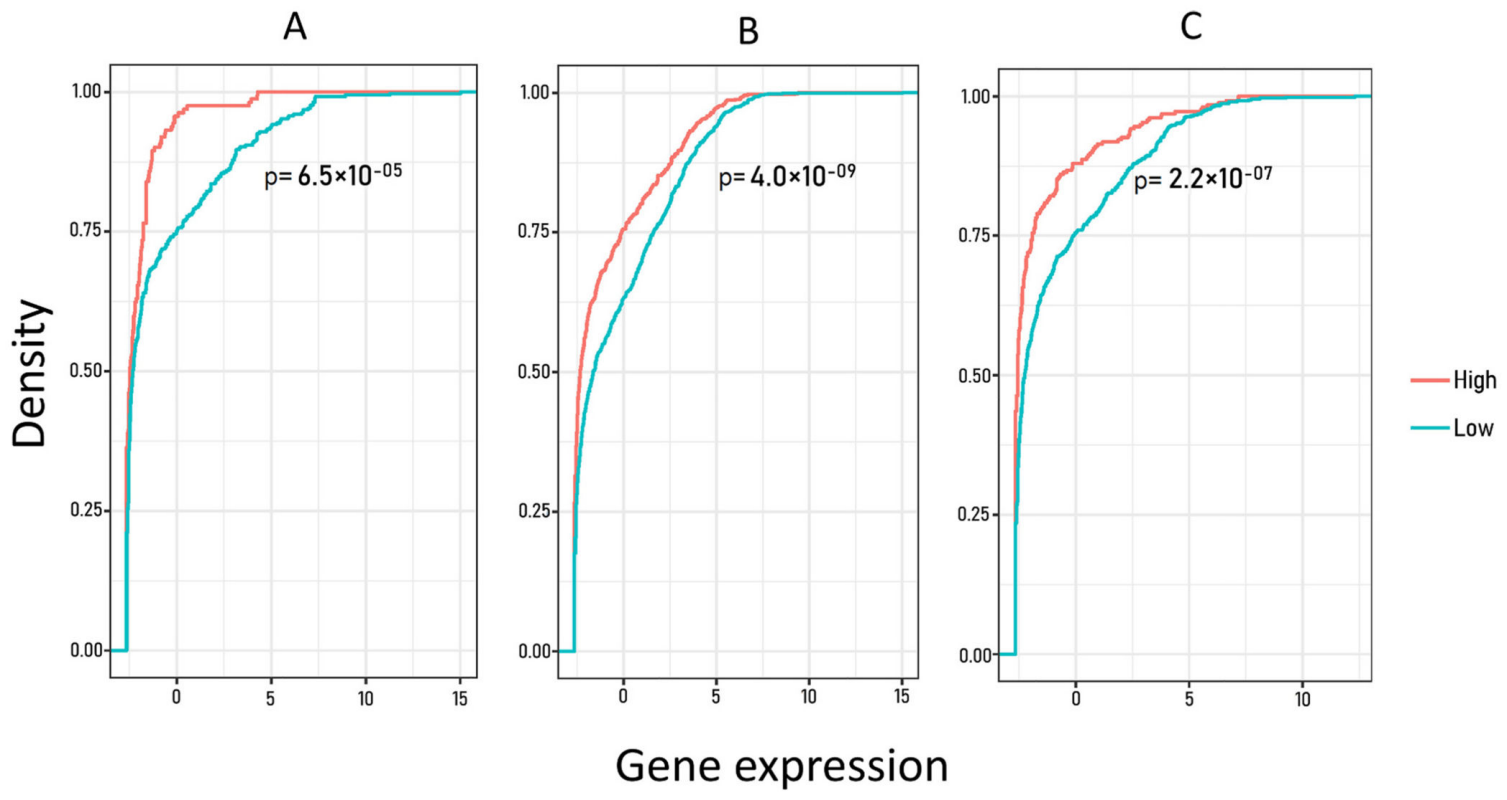
Line graph depicting the correlation between highly and lowly hydroxymethylated genes with their expression. Genes were categorized to highly (red) and lowly (light blue) hydroxymethylated based on the mean 5hmC counts across all muscle samples. The total number of genes containing DhmCs is depicted on the y-axis in four density quantiles from 0 to 100%, while the x-axis shows mean gene expression. The three graphs from left to right represent the correlations between gene expression and 5hmC levels within first exons (A), first introns (B) and promoters (C). *P*-values were calculated based on two-sample, two-sided Kolmogorov-Smirnov test. (For interpretation of the references to colour in this figure legend, the reader is referred to the web version of this article.)

Consistent with our findings above, we identified a negative correlation between 5hmC levels and gene expression in all three comparisons (Fig. 5). Among the most expressed genes in fast muscle, we identified 4 differentially hydroxymethylated genes between muscle and liver. Two DhmCs were located 19,650 and 19,551 bp upstream of the myosin heavy chain fast skeletal muscle (*LOC100706261*) promoter, two DhmCs were located 10,176 and 10,040 bp upstream of the nucleoside diphosphate kinase B (*LOC100697135*) promoter, two DhmCs were found within the 16th exon of *atp2a1l*, one DhmC within the 5th exon of myosin light chain 3 skeletal muscle isoform (*LOC100698429*) and one DhmC within the 1st intron of troponin T fast skeletal muscle isoforms (*LOC100707421*). Surprisingly, all 5hmCs were hypo-hydroxymethylated in muscle compared to liver, except the one that was located closest to the TSS (1318 bp) and within the 1st intron of troponin T fast skeletal muscle isoform (Table 1).

## 3 Discussion

**Fig. 6:**
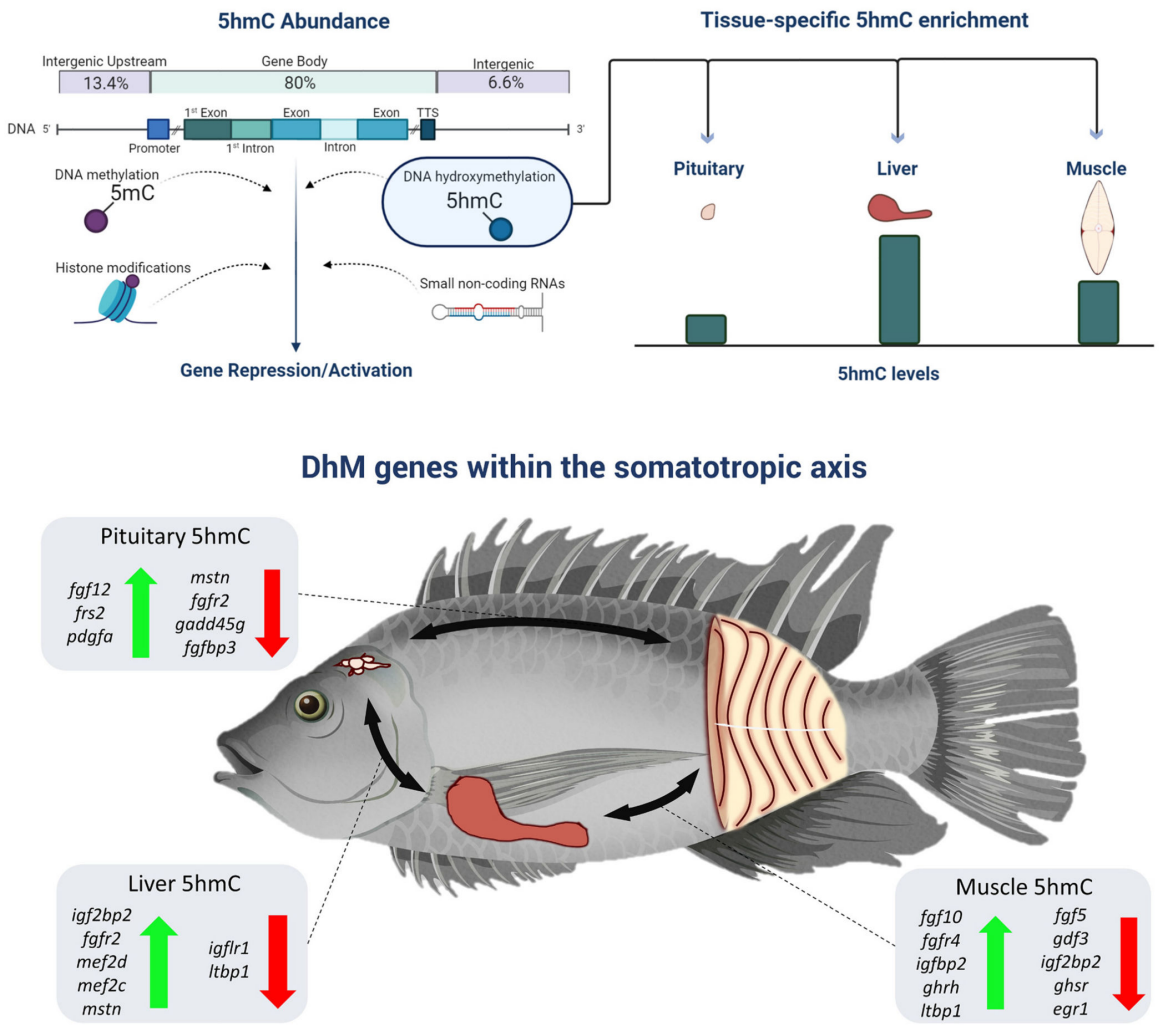
Graphical summary of the main findings of the study. DNA hydroxymethylation was enriched within gene bodies and promoters (80%) compared to intergenic regions (20%). Tissues of the somatotropic axis were differentially hydroxymethylated. The liver had the highest, muscle intermediate, and pituitary the lowest 5hmC levels. Several genes involved in somatic growth were found to be differentially hydroxymethylated among all three comparisons (pituitary – muscle, muscle – liver and liver – pituitary).

This study presents the first DNA hydroxymethylation profiling of the somatotropic axis at single nucleotide resolution in a teleost species. We compared the 5hmC levels among tissues and identified the liver as the tissue with the highest, the muscle with intermediate and the pituitary with the lowest 5hmC levels (Fig. 6). In contrast to these results, global 5hmC levels of the muscle and liver measured by liquid chromatography mass spectrometry (LC/MS) in male zebrafish (*Danio rerio*) revealed that muscle had higher 5hmC levels than liver [44]. Similarly, 5hmC quantification by LC/MS in muscle and liver of mice, showed higher 5hmC levels in muscle than liver [45]. These results can potentially be explained either by species-specific 5hmC patterns or by the methods used, i.e. reduced representation of the genome using RRHP, compared to global 5hmC quantification using LC/MS. The low 5hmC content in pituitary gland in Nile tilapia is in accordance with previous findings in mice [45]. Indeed, reports have demonstrated that high 5hmC levels in brain are associated with tissue-specific neuronal functions [46,47], compared to pituitary, which is responsible for hormone secretion.

Based on the functional enrichment analysis of hyper-hydroxymethylated genes in muscle compared to liver, we identified several genes involved in ionotropic and metabotropic glutamate receptor activity. These genes were associated with all three types of ionotropic glutamate receptors, namely, the *N*-methyl-d-aspartate (NMDA), the kainate and the a-amino-3-hydroxy-5-methyl-4-isoxazolepropionic acid receptors while their functions were mostly associated with fast synaptic transmission and the modulation of synaptic responses [48]. A previous study using C2C12 mouse skeletal myoblasts has shown that NMDA receptors play a major role in calcium influx and myoblast fusion, which are fundamental processes for muscle growth [49]. Furthermore, we also identified several differentially hydroxymethylated metabotropic glutamate receptors (*grm1, grm2, grm4, grm7* and *grm8*) between muscle and liver, which play critical roles in cell survival, metabolism and proliferation through their association with the PI3 kinase and the initiation of the PI3K-Akt-mTOR signaling pathway [50]. Glutamate receptors were found to be under positive selection across several domesticates [51], including fish [52]. Particularly in sea bass (*Dicentrarchus labrax*), the identification of genetic mutations after many generations in captivity was found to be overlapping with early changes in DNA methylation profiles during domestication, suggesting that epimutations potentially drive genome evolution [53]. Here, we identified significantly hydroxymethylated ionotropic and metabotropic glutamate receptors within the somatotropic axis, which supports the functional importance of these genes in somatic growth.

Finally, we identified several DhmCs within the receptor-type tyrosine-protein phosphatase N2 (*ptprn2*) gene, which is expressed in nervous and endocrine cells and it is associated with lipid metabolism and insulin secretion [54,55]. Previous studies have shown that *ptprn2* is largely associated with somatic growth, and regulated by epigenetic modifications in various biological processes including hepatocellular carcinomas [56], breast cancer [57], as well as prenatal growth patterns and birthweight in humans [58]. Therefore, it is likely that DNA hydroxymethylation may modulate somatic growth indirectly, through the regulation of genes related to neuromuscular and neurohepatic signals.

Growth hormone release depends on three hypothalamic factors, namely, growth hormone releasing hormone (GHRH), somatostatin (SST) and ghrelin multifaceted hormone (GHRL) [59,60]. The latter is stimulated by receptors, such as the growth hormone secretagogue receptor (*ghsr*), which was found to be hyper-hydroxymethylated in the liver, compared to the muscle and pituitary. *Ghsr* encodes for a tissue-specific hormone receptor that is commonly found across the somatotropic axis and regulates energy homeostasis, fat distribution and the expression of genes involved in lipid metabolism and total body weight [61,62]. Notably, among the differentially hydroxymethylated genes, we also identified a hyper-5hmC (higher 5hmC level in muscle than liver) within the promoter of the growth hormone *ghrh* as well as several hyper-5hmCs (higher 5hmC levels in muscle and pituitary than liver) within predicted genes coding for somatostatin in Nile tilapia (*loc102077970* – somatostatin-1; *loc100698045* – somatostatin-1B; *loc100694069* – somatostatin-2), known also as growth hormone-inhibiting hormone. Additionally, we identified differentially hydroxymethylated genes that play critical roles in growth hormone signaling and are downstream regulators of somatic growth, such as the insulin-like growth factor binding proteins *igfbp2, igf2bp2* and *igfbp5* and the receptor *igflr1*. In particular, hyper-5hmCs (higher 5hmC levels in muscle than liver) were located within *igfbp2* and the 29th exon of *igflr1* while hypo-5hmCs (lower 5hmC levels in muscle than liver) were located within igfbp5, *igf2bp2* and the 32nd intron *igflr1* (Table S9A). The same genes were also differentially hydroxymethylated between liver and pituitary. A hyper-5hmC (higher 5hmC levels in liver than pituitary) was found within *igf2bp2*, and a hypo-5hmC (lower 5hmC levels in liver than pituitary) was located within *igflr1*. Considering that epigenetic modifications drive tissue-specific gene expression, it is likely that differentially hydroxymethylated cytosines can contribute towards the expression of insulin-like growth factor receptor and binding protein genes. These results suggest a link between DNA hydroxymethylation and the function of the somatotropic axis (Fig. 6).

Interestingly, several genes were both hyper- and hypo-hydroxymethylated between tissues due to the localization of unique DhmCs across their gene body or their proximal regions. For example, the above-mentioned *ptprn2* gene was highly hydroxymethylated in liver (compared to muscle) across the 1st, 3rd and 8th introns as well as the 5th exon, but a hypo-hydroxymethylated cytosine (low 5hmC levels in liver compared to muscle) was also present in the 10th intron. Similar patterns were observed in other genes, including the multiple epidermal growth factor-like domains protein 10 (*mefg10*) and insulin receptor (*insr*), methyltransferases such as *mettl21c* and *mettl4*, adhesion G protein-coupled receptors (*adgrf4, adgrl2, adgrl3, adgrb1*) and several transcription factors (*atf2, atf3, ebf3, grhl1, gtf3c1, gtf3c3, hsf2, sox4* and *runx2*). Taking into consideration the enrichment of 5hmCs within introns and their positional dynamics between tissues, these findings could potentially explain tissue-specific gene expression through the regulation of alternative promoters or splicing junctions. Indeed, recent studies have shown that DNA hydroxymethylation is involved in alternative splicing [63,64] and that positional dynamics of 5hmCs within or in proximal distance of genes result in expression changes [35].

Additionally, studies in mammals and mammalian cells have shown that DNA hydroxymethylation is positively correlated to gene expression and is present in gene bodies of actively transcribed genes [65–67]. Here, even though the correlations of gene expression in the muscle and 5hmC levels were generally moderate (ρ = 0.47–0.63), we found that 5hmCs display a positive correlation with distance from TSSs; the farther the 5hmCs from a TSS, the higher the expression of genes. Although, this correlation included both upstream and downstream 5hmCs from the TSS, 80% of those 5hmCs were located within gene bodies. Therefore, this correlation was primarily affected by 5hmCs within the downstream region of TSSs. Frequently, first introns and exons are positively associated with gene regulation [41–43]. By comparing muscle gene expression and 5hmC levels of promoters, first introns and first exons, we determined that 5hmCs in close proximity to TSSs had an overall negative correlation with gene transcription. However, a close examination of the top 30 most expressed genes in muscle and their hydroxymethylation status revealed that the only hyper-5hmC in muscle compared to liver was also the closest one to the TSS of *troponin T fast skeletal muscle isoform*. Several hypo-5hmCs (lower 5hmC levels in muscle than liver) were found at a distance of 10–19 kbp upstream from the promoter as well as within the open reading frames of highly expressed genes in the muscle but none of them were located within the first intron or exon. These findings suggest that the position of 5hmCs within gene features plays indeed a crucial role in gene repression or activation. The overall negative correlation between 5hmCs and gene expression in our study is likely attributed to the presence of other epigenetic mechanisms that were not profiled here, such as cytosine methylation, histone modifications and non-coding RNAs. Although, high 5hmC levels are usually correlated with gene activation, there is also evidence of 5hmCs being involved in the repression of genes such as the insulin-degrading enzyme (IDE) in depressed individuals [68] and polycomb groups of developmental regulators [66]. In human, there is a high overlap (85.6%) between 5mCs and 5hmCs [69]. Previously, high 5mC levels within the first intron of genes have been strongly associated to low levels of expression across multiple species and tissues [43]. Considering the potential overlap between these two cytosine modifications, their overall contrasting roles in gene regulation, their relative abundance (5mCs are estimated to be 14-fold more abundant than 5hmCs in mammalian embryonic stem cells [28]), and the moderate negative correlation between substantially hydroxymethylated genes and their expression, we propose that the presence of 5hmCs throughout the genome plays a balancing role in gene regulation.

## 4 Conclusions

Somatic growth and normal development are regulated by a complex molecular cascade that is affected by several mechanisms, including epigenetic modifications. Here, we show that DNA hydroxymethylation is present throughout the genome, is characterized by tissue-specific profiles and is highly enriched within gene bodies (Fig. 6).

Several genes that are both directly or indirectly associated with functions such as growth hormone release, insulin-like growth factor binding, liver metabolism and somatic growth were found to be differentially hydroxymethylated between pituitary, liver and muscle. We identified a moderate but significant negative correlation between gene expression and 5hmC levels in muscle. However, one of the most expressed genes in the muscle (*troponin T fast skeletal muscle*) contained a single hyper-5hmC (higher 5hmC levels in the muscle than liver) within the first intron, revealing a positive correlation between DNA hydroxymethylation and gene expression. The high overlap between 5hmCs and 5mCs and their overall contrasting roles in gene transcription pinpoints towards a dynamic interrelationship with DNA hydroxymethylation acting as a balancing factor in gene regulation within tissues. The identification of tissue-specific epigenetic modifications within the somatotropic axis adds an additional layer of complexity to our current knowledge about the regulation of growth.

## 5 Materials and methods

### 5.1 Experimental design and sampling

Female wild Nile tilapia mouthbrooding fertilized eggs were captured using traditional fishing nets and traps at the river Nile in Luxor, Egypt. At 9 days post-hatching, the larvae were transported to our research facilities in Bodø, Norway. A total of 14 batches of juveniles originating from 14 different wild females were tagged and used as the base population (F0) for our Nile tilapia breeding program. These Nile tilapia were reared in a recirculating aquaculture system (pH = 7.6, oxygen saturation = 100%, temperature = 28 °C and photoperiod adjusted at 11:13 dark:light) and fed ad libitum with 0.15–0.8 mm Amber Neptun pellets (Skretting, Norway) as previously described [70]. After a successful reproduction cycle in captivity, we obtained the F1 generation. These fish were PIT-tagged and reared for 8 months in common garden to minimize the effect of environmental factors. For the samples used in this study, 5 full-sib F1 males were randomly selected from the tank. We focused on Nile tilapia males because they are preferred for fish farming, since they grow faster and larger than females. The fish were euthanized by immersion for 3 min in 10 L of water containing 15 mL clove oil pre-mix, consisting of pure clove oil (Sigma Aldrich, USA) and 96% ethanol at a 1:9 ratio. Fast (white) muscle, liver and pituitary gland were carefully dissected, snap-frozen in liquid nitrogen and stored at – 80 °C. In particular, fast muscle was extracted from a 4 mm cross-section at 0.7 standard length. The samples were taken above the lateral line from the left upper white muscle mass. Liver samples were collected from the left lobe and around the entry point of the portal vein. The pituitary gland was extracted by cutting open the skull, removing the brain from the dorsal side and picking the pituitary out with forceps. Information regarding the measurements (weight, total and standard length) of the fish can be found in Table 2.

### 5.2 DNA extraction and RRHP library preparation

DNA extraction was carried out using the Quick-DNA miniprep plus kit (Zymo Research, USA) according to the manufacturer’s instructions. Quantification and integrity control of the extracted DNA was performed using Qubit 3.0 fluorometer and the double-stranded DNA high sensitivity assay kit (ThermoFisher Scientific, USA) and Tapestation 2200 (Agilent Technologies, USA), respectively.

Reduced representation of 5-hydroxymethylcytosine profiling (RRHP) was performed according to manufacturer’s protocol (Zymo Research). The preparation of RRHP libraries was performed synchronously for all tissues and replicates. The starting DNA concentration was 150 ng diluted in 10 μL DNAse and RNAse free water. Sequencing was performed using Nord University’s inhouse NextSeq500 (Illumina, USA) and all libraries were pooled with equal volumes. Finally, they were distributed among three High-Output kit v2.5 150 cycle flow cells, yielding approximately 360 million single-end reads.

### 5.3 RRHP bioinformatic pipeline

Raw reads were trimmed for adapters using trim_galore v0.4.4 and quality check was performed using MultiQC. The software Bowtie v0.12.8 was used for the alignment of high quality and trimmed reads to the latest Nile tilapia reference genome (NCBI assembly GCA_001858045.3) [71] with the following parameters: -S -v 1 -n 1 -m 3 –strata –best. The chromosome, position and strand information from reads that start with CCGG were extracted in text files for each sample. These files were used as input into R, where a count matrix was created by summing up overlapping chromosomal positions. To minimize the false discovery of 5hmCs the data set was filtered twice, as reported [70]. Briefly, the first filter removed reads for which more than two groups of samples (2 out of 3 tissues) had less than 1 count. For the second filter, the median of the entire dataset was calculated and 5hmC sites were removed when at least one tissue among the three (5 or more samples) had counts equal or less than the median (21 counts). For the comparison of 5hmC levels at single nucleotide resolution between the three tissues, we used the R package limma [72] and the adjusted *p*-values were calculated using the Benjamini-Hochberg correction. Three contrasts were performed comparing the 5hmC levels of i) the muscle against the liver (MvL), ii) the muscle against the pituitary gland (MvP) and iii) the liver against the pituitary gland (LvP).

### 5.4 Functional enrichment analysis

Differentially hydroxymethylated cytosines (DhmCs) were assigned to genes based on their proximal distance to transcription start sites, using the all-in-one Perl script annotatepeaks.pl from HOMER (http://homer.ucsd.edu). Genes associated with DhmCs were considered to be differentially hydroxymethylated. DhmCs located within uncharacterized genes were excluded from further analysis, since they lack information regarding their function. In total, 4590 DhmCs in MvL, 463 DhmCs in MvP and 367 DhmCs in LvP comparisons were annotated within uncharacterized genes. Functional enrichment analysis was performed using gProfiler (version: e99_eg46_p14_f929183) with default parameters for *Oreochromis niloticus*. Multiple testing correction was performed using the suggested tailor-made algorithm g:SCS and the significance threshold was set at 0.05 [73]. The analysis was separated in 6 stages using hyper- and hypo-hydroxymethylated genes per pairwise tissue comparison as well as 3 additional stages for genes that were both hypo- and hyper-hydroxymethylated in a single tissue per pairwise tissue comparison.

### 5.5 RNA extraction, library preparation and sequencing

The same fast muscle samples that were used for the RRHP library preparation were homogenised in DNA/RNA Shield (Zymo Research) using ZR bashing beads lysis tubes (Zymo Research). Homogenization was carried out using a Precellys 24 homogenizer (Bertin Instruments, France) with 2 cycles at 5000 rpm and 20 s duration. RNA extraction was performed using the Quick-RNA miniprep kit (Zymo Research) following the manufacturer’s protocol for samples stored in DNA/RNA Shield. The Ribo-Zero Gold rRNA removal kit (Illumina) was used to ensure the removal of ribosomal RNA. RNA quality was determined using the spectrophotometer NanoDrop ND 1000 (ThermoFisher Scientific), while its concentration and integrity was measured with an RNA High-sensitivity screentape on a Tapestation 2200 (Agilent Technologies). For the library preparation, the NEBNEXT Ultra II Directional RNA library prep kit for Illumina (NEB, USA) was used following the manufacturer’ s instructions and recommendations. Each sample was tagged with a unique barcode and the libraries were pooled at equimolar concentrations. The pool was sequenced at the Norwegian Sequencing Centre on a HiSeq 4000 lane (Illumina) in paired-end 150 bp mode.

### 5.6 Bioinformatic analysis of RNA-seq data

The sequencing data obtained above (section 5.5) were trimmed using BBDuk of the BBTools Suite (v. 38.22-0, Joint Genome Institute), which decontaminates using Kmers. Adapters were trimmed as per default (ktrim = r, k = 23, mink = 11, hdist = 1, tpe, tbo) and quality trimmed for both sides of the read with the Phred threshold set to 20, while reads less than 23 bp after trimming to Q20 were discarded. An index was built with the HISAT2 (v. 2.1.0) program [74] using the Nile tilapia reference genome above and trimmed reads were aligned by the core function of HISAT2 with RNA strandness set to FR and reported alignments tailored for downstream transcript assemblers. Alignments were sorted by samtools (v. 1.9) and a summarized report for all samples was generated using the MultiQC software [75]. Calculation of count numbers for each gene was performed by featureCounts (v 1.6.2) [76] of the Subread package [77] using the gff3 genome annotation (NCBI, ref_O_niloticus_UMD_NMBU_top_level.gff3). Chimeric fragments were excluded, duplicates were ignored, only primary alignments were counted and fragments were allowed to match more than one metafeature. Further analysis were performed in R (v. 3.6.1) [78] and Rstudio (1.2.1335) [79]. The DESeq2 package [80] was used for applying a regularized log transformation to the data in order to minimize differences between small counts and normalize according to the library size in an unbiased manner by prior information.

For each cytosine with 5hmC information, the mean of 5hmC counts across all five biological replicates was used together with the distance of each C from the TSS. Each C corresponded to a single expressed gene, the value of which was estimated based on the mean of gene expression counts across all five biological replicates. In other words, a value of gene expression could be attributed to more than one C. Principal component analysis and visualizations were performed with the Facto-MineR (v. 1.42) and factoextra (v. 1.0.5) packages.

## Supplementary Material

Supplementary data to this article can be found online at https://doi.

Supplementary Material

## Figures and Tables

**Table 1:** List of DhmCs between muscle and liver, found within the most expressed genes in muscle. Positive and negative logFC values correspond to hyper- and hypo-hydroxymethylation in muscle compared to liver, respectively.

Chromosome	Position	LogFC	Annotation	Dist. to TSS	Gene Symbol	Description
NC_031972.2	3,582,578	0.70	Intron (1/11)	1318	*LOC100707421*	troponin T fast skeletal muscle isoforms
NC_031973.2	16,443,052	−1.51	Exon (16/24)	7879	*LOC100706607*	sarcoplasmic/endoplasmic reticulum calcium ATPase 1
NC_031986.2	22,143,050	−1.53	Exon (5/6)	2333	*LOC100698429*	myosin light chain 3 skeletal muscle isoform
NC_031969.2	34,504,991	−1.74	Intergenic	10,176	*LOC100697135*	nucleoside diphosphate kinase B
NC_031969.2	34,505,127	−1.94	Intergenic	10,040	*LOC100697135*	nucleoside diphosphate kinase B
NC_031973.2	16,442,970	− 2.01	Exon (16/24)	7797	*LOC100706607*	sarcoplasmic/endoplasmic reticulum calcium ATPase 1
NC_031969.2	23,538,241	− 2.72	Intergenic	19,650	*LOC100698429*	myosin heavy chain fast skeletal muscle
NC_031969.2	23,538,340	− 3.18	Intergenic	19,551	*LOC100698429*	myosin heavy chain fast skeletal muscle

**Table 2:** Sampling information. The list includes measurements of weight, total and standard length of all the individuals used for the current study (*n* = 5) as well as their family and passive integrated transponder tag information.

Sample ID	Family	PIT	Weight (g)	Total length (cm)	Standard length (cm)
M1	f1c9	439E4	1001	37.1	29.7
M2	f1c9	440D7	683	33.9	26.8
M3	f1c9	4296F	1298	37.9	29.1
M4	f1c9	4485C	915	35.9	28
M5	f1c9	44BE5	708	34.2	26.4

## Data Availability

The DNA hydroxymethylation dataset of this study is available in the SRA (NCBI) repository, under the accession number PRJNA665628. The RNA sequencing dataset of this study is available in the GEO (NCBI) repository, under the accession number GSE158910.
