# Supplementary material for: Epigenetic mapping of the somatotropic axis in Nile tilapia reveals differential DNA hydroxymethylation marks associated with growth
Source: Genomics. Author manuscript; Available in PMC 2021 Jul 19. (PMC7611323; doi:10.1016/j.ygeno.2021.06.037)
Supplement: Supplementary Material [file EMS129618-supplement-Supplementary_Material.pdf]

Supplementary Material

Title: Epigenetic mapping of the somatotropic axis in Nile tilapia reveals differential DNA hydroxymethylation marks associated with growth

Authors

Ioannis Konstantinidis[1], Dafni Anastasiadi[2,3], Pål Sætrom[1,4,5,6,7], Artem V. Nedoluzhko[1], Robin Mjelle[1,4], Tomasz Podgorniak[1], Francesc Piferrer[3], Jorge M. O. Fernandes[1*]

Affiliations

[1]Faculty of Biosciences and Aquaculture, Nord University, Bodø, Norway.

[2]The New Zealand Institute for Plant and Food Research, Nelson, New Zealand.

[3]Institut de Ciències del Mar, Spanish National Research Council (CSIC), Barcelona, Spain.

[4]Department of Clinical and Molecular Medicine, Norwegian University of Science and Technology, Trondheim, Norway.

[5]Department of Computer Science, Norwegian University of Science and Technology, Trondheim, Norway.

[6]Bioinformatics core facility-BioCore, Norwegian University of Science and Technology, Trondheim, Norway.

[7]K.G. Jebsen Center for Genetic Epidemiology, Norwegian University of Science and Technology, Trondheim, Norway.

*Corresponding author: Jorge M. O. Fernandes, email: jorge.m.fernandes@nord.no

Supplementary tables

Table S1. List of differentially hydroxymethylated cytosines (DhmCs) among all pairwise tissue comparisons. Their location within the Nile tilapia genome (NCBI assembly GCA_001858045.3) is depicted in the first three columns (Chromosome, Position and Strand). Gene symbols and their annotation are based on the output by HOMER. Adjusted p-values for each pairwise tissue comparison: muscle compared to pituitary (MvP), liver compared to pituitary (LvP) and muscle compared to liver (MvL).

Chromosome	Position	Strand	Gene Symbols	Annotation	adj.P.Val (MvP)	adj.P.Val (LvP)	adj.P.Val (MvL)
NC_031973.2	19766539	-	*gjd3*	exon	4.47E-02	8.37E-03	5.69E-05
NC_031979.2	27773557	-	*LOC102076435*	lncRNA	3.35E-02	7.15E-04	8.14E-04
NC_031982.2	28743745	-	*pard3ab*	Intron	1.48E-02	2.50E-03	1.44E-05
NC_031974.2	3115354	+	*LOC112847764*	lncRNA	4.37E-02	9.84E-03	5.76E-05

Table S2. List of significantly enriched molecular functions and biological processes. The list is created based on all hypo-hydroxymethylated genes in muscle compared to liver (gProfiler, multiple testing correction g:SCS, q<0.05).

**Source**	**Term name**	**Term ID**	**adjusted p value**
GO:MF	protein binding	GO:0005515	9.7E-10
GO:MF	transcription regulator activity	GO:0140110	3.2E-05
GO:MF	DNA-binding transcription factor activity	GO:0003700	1.3E-04
GO:MF	enzyme binding	GO:0019899	1.6E-04
GO:MF	GTPase binding	GO:0051020	1.0E-03
GO:MF	Ras GTPase binding	GO:0017016	3.0E-03
GO:MF	binding	GO:0005488	3.3E-03
GO:MF	chromatin binding	GO:0003682	3.8E-03
GO:MF	small GTPase binding	GO:0031267	4.1E-03
GO:MF	GTPase activator activity	GO:0005096	3.2E-02
GO:MF	sequence-specific DNA binding	GO:0043565	3.9E-02
GO:BP	anatomical structure development	GO:0048856	6.8E-17
GO:BP	developmental process	GO:0032502	9.4E-17
GO:BP	multicellular organism development	GO:0007275	6.0E-16
GO:BP	system development	GO:0048731	3.3E-13
GO:BP	multicellular organismal process	GO:0032501	1.0E-12
GO:BP	regulation of cellular process	GO:0050794	5.0E-11
GO:BP	regulation of biological process	GO:0050789	6.8E-11
GO:BP	regulation of RNA metabolic process	GO:0051252	4.3E-09
GO:BP	animal organ development	GO:0048513	4.9E-09
GO:BP	regulation of nucleic acid-templated transcription	GO:1903506	6.6E-09
GO:BP	regulation of RNA biosynthetic process	GO:2001141	6.6E-09
GO:BP	regulation of cellular metabolic process	GO:0031323	1.3E-08
GO:BP	biological regulation	GO:0065007	1.4E-08
GO:BP	regulation of nucleobase-containing compound metabolic process	GO:0019219	1.4E-08
GO:BP	regulation of nitrogen compound metabolic process	GO:0051171	1.8E-08
GO:BP	regulation of cell communication	GO:0010646	2.2E-08
GO:BP	regulation of transcription, DNA-templated	GO:0006355	2.4E-08
GO:BP	anatomical structure morphogenesis	GO:0009653	2.8E-08
GO:BP	cell differentiation	GO:0030154	3.4E-08
GO:BP	regulation of biosynthetic process	GO:0009889	3.8E-08
GO:BP	regulation of cellular biosynthetic process	GO:0031326	3.8E-08
GO:BP	regulation of signaling	GO:0023051	3.9E-08
GO:BP	regulation of primary metabolic process	GO:0080090	4.0E-08
GO:BP	cell surface receptor signaling pathway	GO:0007166	4.1E-08
GO:BP	tissue development	GO:0009888	4.2E-08
GO:BP	regulation of macromolecule metabolic process	GO:0060255	5.3E-08
GO:BP	cellular developmental process	GO:0048869	6.7E-08
GO:BP	regulation of macromolecule biosynthetic process	GO:0010556	6.9E-08
GO:BP	nucleic acid-templated transcription	GO:0097659	7.1E-08

GO:BP	cell communication	GO:0007154	7.2E-08
GO:BP	regulation of metabolic process	GO:0019222	8.9E-08
GO:BP	RNA biosynthetic process	GO:0032774	9.0E-08
GO:BP	regulation of signal transduction	GO:0009966	9.2E-08
GO:BP	regulation of cellular macromolecule biosynthetic process	GO:2000112	2.8E-07
GO:BP	transcription, DNA-templated	GO:0006351	2.8E-07
GO:BP	regulation of gene expression	GO:0010468	2.9E-07
GO:BP	signaling	GO:0023052	4.2E-07
GO:BP	RNA metabolic process	GO:0016070	3.7E-06
GO:BP	signal transduction	GO:0007165	4.6E-06
GO:BP	nervous system development	GO:0007399	6.1E-06
GO:BP	cell development	GO:0048468	6.2E-06
GO:BP	regulation of response to stimulus	GO:0048583	1.1E-05
GO:BP	neurogenesis	GO:0022008	1.4E-05
GO:BP	generation of neurons	GO:0048699	1.8E-05
GO:BP	cellular response to stimulus	GO:0051716	2.0E-05
GO:BP	macromolecule biosynthetic process	GO:0009059	2.3E-05
GO:BP	neuron differentiation	GO:0030182	4.7E-05
GO:BP	anatomical structure formation involved in morphogenesis	GO:0048646	6.0E-05
GO:BP	nucleobase-containing compound biosynthetic process	GO:0034654	6.5E-05
GO:BP	cellular macromolecule biosynthetic process	GO:0034645	1.2E-04
GO:BP	cell morphogenesis involved in differentiation	GO:0000904	1.3E-04
GO:BP	aromatic compound biosynthetic process	GO:0019438	1.4E-04
GO:BP	connective tissue development	GO:0061448	1.4E-04
GO:BP	skeletal system development	GO:0001501	1.6E-04
GO:BP	heterocycle biosynthetic process	GO:0018130	2.3E-04
GO:BP	axonogenesis	GO:0007409	2.9E-04
GO:BP	cartilage development	GO:0051216	5.1E-04
GO:BP	cell morphogenesis involved in neuron differentiation	GO:0048667	6.6E-04
GO:BP	regulation of intracellular signal transduction	GO:1902531	8.9E-04
GO:BP	axon development	GO:0061564	9.6E-04
GO:BP	organic cyclic compound biosynthetic process	GO:1901362	9.9E-04
GO:BP	circulatory system development	GO:0072359	2.0E-03
GO:BP	intracellular signal transduction	GO:0035556	2.8E-03
GO:BP	cell part morphogenesis	GO:0032990	3.3E-03
GO:BP	neuron development	GO:0048666	3.4E-03
GO:BP	response to stimulus	GO:0050896	3.5E-03
GO:BP	gene expression	GO:0010467	3.6E-03
GO:BP	epithelium development	GO:0060429	3.6E-03
GO:BP	neuron projection morphogenesis	GO:0048812	3.9E-03
GO:BP	plasma membrane bounded cell projection morphogenesis	GO:0120039	3.9E-03
GO:BP	cell projection morphogenesis	GO:0048858	3.9E-03
GO:BP	cellular component morphogenesis	GO:0032989	4.3E-03
GO:BP	positive regulation of nitrogen compound metabolic process	GO:0051173	4.4E-03
GO:BP	positive regulation of cellular metabolic process	GO:0031325	4.7E-03
GO:BP	positive regulation of metabolic process	GO:0009893	5.6E-03

GO:BP	cell morphogenesis	GO:0000902	6.2E-03
GO:BP	positive regulation of macromolecule metabolic process	GO:0010604	6.8E-03
GO:BP	animal organ morphogenesis	GO:0009887	1.9E-02
GO:BP	neuron projection development	GO:0031175	2.0E-02
GO:BP	axon guidance	GO:0007411	2.1E-02
GO:BP	transcription by RNA polymerase II	GO:0006366	2.5E-02
GO:BP	cellular biosynthetic process	GO:0044249	2.5E-02
GO:BP	sensory epithelium regeneration	GO:0070654	2.7E-02
GO:BP	epithelium regeneration	GO:1990399	2.7E-02
GO:BP	neuron projection guidance	GO:0097485	2.7E-02
GO:BP	muscle structure development	GO:0061061	3.4E-02
GO:BP	enzyme linked receptor protein signaling pathway	GO:0007167	3.4E-02
GO:BP	central nervous system development	GO:0007417	3.4E-02
GO:BP	regulation of transcription by RNA polymerase II	GO:0006357	4.2E-02
GO:BP	chordate embryonic development	GO:0043009	4.5E-02
GO:CC	nucleus	GO:0005634	3.5E-04
HP	Joint hyperflexibility	HP:0005692	2.7E-02

Table S3. List of significantly enriched molecular functions and biological processes. The list is created based on all hyper-hydroxymethylated genes in muscle compared to liver (gProfiler, multiple testing correction g:SCS, q<0.05).

Source	Term name	Term ID	adjusted p value
GO:MF	ionotropic glutamate receptor activity	GO:0004970	1.8E-03
GO:MF	glutamate receptor activity	GO:0008066	1.8E-03
GO:MF	sequence-specific DNA binding	GO:0043565	1.8E-02
GO:MF	molecular transducer activity	GO:0060089	4.9E-02
GO:MF	signaling receptor activity	GO:0038023	4.9E-02
GO:BP	cell surface receptor signaling pathway	GO:0007166	1.5E-03
GO:BP	system development	GO:0048731	3.2E-03
GO:BP	ionotropic glutamate receptor signaling pathway	GO:0035235	4.5E-03
GO:BP	regulation of nucleic acid-templated transcription	GO:1903506	5.6E-03
GO:BP	regulation of RNA biosynthetic process	GO:2001141	5.6E-03
GO:BP	regulation of RNA metabolic process	GO:0051252	6.8E-03
GO:BP	regulation of transcription, DNA-templated	GO:0006355	7.8E-03
GO:BP	multicellular organism development	GO:0007275	8.1E-03
GO:BP	regulation of nucleobase-containing compound metabolic process	GO:0019219	9.5E-03
GO:BP	multicellular organismal process	GO:0032501	9.6E-03
GO:BP	anatomical structure development	GO:0048856	1.7E-02
GO:BP	developmental process	GO:0032502	1.9E-02
GO:BP	regulation of macromolecule biosynthetic process	GO:0010556	2.0E-02
GO:BP	glutamate receptor signaling pathway	GO:0007215	2.3E-02
GO:BP	organic cyclic compound biosynthetic process	GO:1901362	2.3E-02
GO:BP	aromatic compound biosynthetic process	GO:0019438	2.3E-02
GO:BP	regulation of cellular macromolecule biosynthetic process	GO:2000112	2.4E-02
GO:BP	nucleobase-containing compound biosynthetic process	GO:0034654	3.1E-02
GO:BP	regulation of cellular biosynthetic process	GO:0031326	3.3E-02
GO:BP	heterocycle biosynthetic process	GO:0018130	3.5E-02
GO:BP	regulation of biosynthetic process	GO:0009889	3.9E-02
GO:BP	nucleic acid-templated transcription	GO:0097659	4.4E-02
GO:BP	RNA biosynthetic process	GO:0032774	4.8E-02
GO:BP	animal organ development	GO:0048513	5.0E-02

Table S4. List of significantly enriched molecular functions and biological processes. The list is created based on 773 genes that contained hyper-hydroxymethylcytosines in both muscle and liver (gProfiler, multiple testing correction g:SCS, q<0.05).

Source	Term name	Term ID	adjusted p value
GO:MF	ionotropic glutamate receptor activity	GO:0004970	1.3E-03
GO:MF	glutamate receptor activity	GO:0008066	1.3E-03
GO:MF	signaling receptor activity	GO:0038023	6.6E-03
GO:MF	molecular transducer activity	GO:0060089	6.6E-03
GO:MF	transmitter-gated channel activity	GO:0022835	2.1E-02
GO:MF	transmitter-gated ion channel activity	GO:0022824	2.1E-02
GO:MF	gated channel activity	GO:0022836	3.3E-02
GO:MF	extracellular ligand-gated ion channel activity	GO:0005230	4.6E-02
GO:BP	regulation of biological process	GO:0050789	1.5E-04
GO:BP	biological regulation	GO:0065007	5.0E-04
GO:BP	regulation of cellular process	GO:0050794	7.6E-04
GO:BP	ionotropic glutamate receptor signaling pathway	GO:0035235	4.2E-03
GO:BP	signaling	GO:0023052	9.1E-03
GO:BP	glutamate receptor signaling pathway	GO:0007215	1.4E-02
GO:BP	cell communication	GO:0007154	1.7E-02
GO:BP	signal transduction	GO:0007165	2.0E-02
GO:BP	cell surface receptor signaling pathway	GO:0007166	2.0E-02
GO:BP	response to stimulus	GO:0050896	2.7E-02
GO:BP	cellular response to stimulus	GO:0051716	4.3E-02
GO:CC	cell periphery	GO:0071944	2.9E-02
GO:CC	postsynaptic membrane	GO:0045211	3.9E-02
GO:CC	synaptic membrane	GO:0097060	3.9E-02

Table S5. List of significantly enriched molecular functions and biological processes. The list is created based on all hyper-hydroxymethylated genes in muscle compared to pituitary (gProfiler, multiple testing correction g:SCS, q<0.05).

Source	Term name	Term ID	adjusted p value
GO:MF	protein binding	GO:0005515	3.3E-03
GO:BP	tissue morphogenesis	GO:0048729	4.8E-03
GO:BP	establishment of organelle localization	GO:0051656	1.3E-02
GO:BP	anatomical structure development	GO:0048856	1.4E-02
GO:BP	morphogenesis of an epithelium	GO:0002009	1.6E-02
GO:BP	developmental process	GO:0032502	1.6E-02
GO:BP	ameboidal-type cell migration	GO:0001667	1.7E-02
GO:BP	signaling	GO:0023052	2.1E-02
GO:BP	establishment of localization in cell	GO:0051649	3.0E-02
GO:BP	embryonic eye morphogenesis	GO:0048048	3.1E-02
GO:BP	cell communication	GO:0007154	3.3E-02
GO:BP	cellular response to stimulus	GO:0051716	3.7E-02
GO:BP	regulation of biological process	GO:0050789	4.5E-02

Table S6. List of significantly enriched molecular functions and biological processes. The list is created based on all hypo-hydroxymethylated genes in muscle compared to pituitary (gProfiler, multiple testing correction g:SCS, q<0.05).

Source	Term name	Term ID	adjusted p value
GO:MF	DNA-binding transcription factor activity	GO:0003700	1.8E-02
GO:MF	transcription regulator activity	GO:0140110	4.9E-02
GO:BP	regulation of nucleobase-containing compound metabolic process	GO:0019219	4.8E-04
GO:BP	regulation of RNA metabolic process	GO:0051252	1.3E-03
GO:BP	regulation of transcription, DNA-templated	GO:0006355	1.7E-03
GO:BP	regulation of nitrogen compound metabolic process	GO:0051171	2.6E-03
GO:BP	regulation of RNA biosynthetic process	GO:2001141	2.7E-03
GO:BP	regulation of nucleic acid-templated transcription	GO:1903506	2.7E-03
GO:BP	RNA metabolic process	GO:0016070	3.1E-03
GO:BP	regulation of cellular macromolecule biosynthetic process	GO:2000112	3.4E-03
GO:BP	regulation of cellular biosynthetic process	GO:0031326	4.0E-03
GO:BP	regulation of primary metabolic process	GO:0080090	4.1E-03
GO:BP	regulation of biosynthetic process	GO:0009889	4.6E-03
GO:BP	transcription, DNA-templated	GO:0006351	4.7E-03
GO:BP	regulation of gene expression	GO:0010468	4.9E-03
GO:BP	regulation of macromolecule biosynthetic process	GO:0010556	5.9E-03
GO:BP	nucleic acid-templated transcription	GO:0097659	7.0E-03
GO:BP	RNA biosynthetic process	GO:0032774	7.4E-03
GO:BP	regulation of cellular metabolic process	GO:0031323	7.9E-03
GO:BP	regulation of biological process	GO:0050789	1.6E-02
GO:BP	regulation of macromolecule metabolic process	GO:0060255	1.8E-02
GO:BP	regulation of cellular process	GO:0050794	2.1E-02
GO:BP	regulation of metabolic process	GO:0019222	3.5E-02
GO:CC	nucleus	GO:0005634	1.8E-04
GO:CC	organelle	GO:0043226	2.1E-02
GO:CC	membrane-bounded organelle	GO:0043227	2.8E-02
GO:CC	intracellular organelle	GO:0043229	3.6E-02

Table S7. List of significantly enriched molecular functions and biological processes. The list is created based on all hypo-hydroxymethylated genes in liver compared to pituitary (gProfiler, multiple testing correction g:SCS, q<0.05).

Source	Term name	Term ID	adjusted p value
GO:BP	cell surface receptor signaling pathway	GO:0007166	4.3E-02
GO:BP	axon target recognition	GO:0007412	5.0E-02

Table S8. List of significantly enriched molecular functions and biological processes. The list is created based on all hyper-hydroxymethylated genes in liver compared to pituitary (gProfiler, multiple testing correction g:SCS, $q<0.05$).

Source	Term name	Term ID	adjusted p value
GO:MF	protein binding	GO:0005515	1.1E-03
GO:MF	sequence-specific DNA binding	GO:0043565	1.4E-02
GO:MF	binding	GO:0005488	1.6E-02
GO:MF	DNA-binding transcription factor activity	GO:0003700	4.6E-02
GO:BP	regulation of cellular process	GO:0050794	4.3E-05
GO:BP	regulation of biological process	GO:0050789	1.4E-03
GO:BP	signaling	GO:0023052	4.7E-03
GO:BP	signal transduction	GO:0007165	5.2E-03
GO:BP	biological regulation	GO:0065007	6.8E-03
GO:BP	developmental process	GO:0032502	7.4E-03
GO:BP	skeletal system development	GO:0001501	7.9E-03
GO:BP	cell communication	GO:0007154	1.6E-02
GO:BP	anatomical structure development	GO:0048856	2.4E-02
GO:BP	regulation of cellular metabolic process	GO:0031323	2.6E-02
GO:BP	tissue development	GO:0009888	3.0E-02
GO:BP	regulation of nitrogen compound metabolic process	GO:0051171	3.3E-02
GO:BP	embryo development	GO:0009790	4.0E-02
GO:BP	multicellular organism development	GO:0007275	4.2E-02
GO:BP	regulation of primary metabolic process	GO:0080090	4.9E-02
HP	Optic disc hypoplasia	HP:0007766	4.7E-02
HP	Aplasia/Hypoplasia of the optic nerve	HP:0008058	4.8E-02

Table S9. List of genes associated with somatic growth and found to be both hypo- and hyper-hydroxymethylated within our pairwise comparisons. Each pairwise comparison is separated in muscle vs liver (**A**), muscle vs pituitary (**B**), and liver vs pituitary (**C**). The information provided in the table contains the precise location of 5hmCs in the Nile tilapia genome (Chromosome, Position and Strand), the log fold change (logFC) of DNA hydroxymethylation levels between the corresponding tissues and their annotation (annotated feature, Distance to TSS, transcript, gene symbol and description) based on software HOMER.

Table S9. A)

Chromosome	Position	Strand	logFC	Annotated feature	Distance to TSS	Transcript	Gene symbol	Description	Transcript variant
NC_031965.2	5670962	-	5.049603334	Intergenic	-20551	rna346	megf10	multiple epidermal growth factor-like domains protein 10	
NC_031978.2	33510485	-	4.417522549	intron (rna38385, intron 3 of 27)	33853	rna38387	ltbp1	latent transforming growth factor beta binding protein 1	X6
NC_031969.2	30907456	-	2.954056767	Intergenic	-3202	rna8108	myh	myosin heavy chain fast skeletal muscle	
NC_031965.2	5671232	-	1.874103248	Intergenic	-20821	rna346	megf10	multiple epidermal growth factor-like domains protein 10	
NC_031966.2	2128899	-	1.768056981	Intergenic	9373	rna2695	LOC100707945	fibroblast growth factor 4-like	
NC_031987.2	6074081	-	1.65811139	intron (rna45359, intron 1 of 3)	5440	rna45359	igfbp2	insulin-like growth factor-binding protein 2-B	
NC_031965.2	5665905	-	1.634262271	Intergenic	-15494	rna346	megf10	multiple epidermal growth factor-like domains protein 10	
NC_031965.2	5670881	+	1.51429004	Intergenic	-20470	rna346	megf10	multiple epidermal growth factor-like domains protein 10	
NC_031977.2	26335498	-	1.418476859	TTS (rna34682)	-6413	rna34683	fgf10	fibroblast growth factor 10	
NC_031981.2	7788462	-	1.391773813	intron (rna48441, intron 7 of 7)	19967	rna48446	mdfic	myoD family inhibitor domain-containing protein	
NC_031969.2	30902097	+	1.12920771	intron (rna8108, intron 2 of 40)	2157	rna8108	myh	myosin heavy chain fast skeletal muscle	
NC_031978.2	33448488	+	1.108470798	intron (rna38363, intron 17 of 18)	-28142	rna38382	ltbp1	latent transforming growth factor beta binding protein 1	X1
NC_031970.2	33693923	+	1.104093484	promoter-TSS (rna11647)	81	rna11647	ghrh	growth hormone releasing hormone	X1

NC_031975.2	23692177	+	1.071004923	Intergenic	26394	rna28268	fgfr4	fibroblast growth factor receptor 4	
NC_031972.2	31905043	-	1.002312173	exon (rna18174, exon 4 of 4)	3166	rna18171	LOC100706093	growth arrest and DNA damage-inducible protein GADD45 gamma	
NC_031976.2	17588156	-	0.968945827	exon (rna30286, exon 29 of 36)	12941	rna30287	igflr1	IGF like family receptor 1	
NC_031976.2	38708518	+	0.962106678	intron (rna32474, intron 1 of 8)	393	rna32474	LOC100708478	tropomyosin alpha-3 chain	
NC_031970.2	25461602	+	0.922276273	promoter-TSS (rna10856)	-832	rna10856	mdfic	myoD family inhibitor domain-containing protein	
NC_031972.2	17045981	+	0.898171306	intron (rna17062, intron 9 of 17)	6969	rna17062	LOC100697438	BDNF/NT-3 growth factors receptor	
NC_031966.2	25226315	+	0.844437322	exon (rna4306, exon 3 of 23)	12256	rna4306	pdgfrb	platelet derived growth factor receptor beta	
NC_031965.2	5623306	+	0.782841003	intron (rna346, intron 8 of 17)	27105	rna346	megf10	multiple epidermal growth factor-like domains protein 10	
NC_031983.2	30155064	-	0.774747785	TTS (rna56828)	4537	rna56828	gdf6a	growth/differentiation factor 6-A-like	
NC_031979.2	17717350	+	0.679548098	intron (rna39782, intron 2 of 7)	26611	rna39783	pdgfd	platelet derived growth factor D	X2
NC_031981.2	3278812	+	0.638710167	Intergenic	49439	rna48180	rerg	RAS like estrogen regulated growth inhibitor	X4
NC_031983.2	10433489	+	0.597573992	intron (rna55019, intron 28 of 30)	26183	rna55013	tgfb3	transforming growth factor beta 3	
NC_031978.2	33448861	-	0.593273156	intron (rna38363, intron 17 of 18)	-27769	rna38382	ltbp1	latent transforming growth factor beta binding protein 1	X1
NC_031986.2	23248307	+	-0.82492603	intron (rna64996, intron 1 of 3)	2281	rna64996	igfbp5	insulin-like growth factor-binding protein 5	
NC_031980.2	5954961	+	-0.828481452	Intergenic	-3012	rna42234	ctgf	connective tissue growth factor	
NC_031969.2	30860092	+	-0.856852347	promoter-TSS (rna8101)	-252	rna8100	gas7	growth arrest-specific protein 7	
NC_031987.2	6091303	+	-0.861075799	intron (rna45359, intron 3 of 3)	22425	rna45360	igfbp5	insulin like growth factor binding protein 5	
NC_031970.2	25429102	-	-0.94728757	intron (rna10853, intron 5 of 18)	-33332	rna10856	mdfic	myoD family inhibitor domain-containing protein	
NC_031986.2	31401855	+	-0.951473703	intron (rna65714, intron 1 of 17)	3582	rna65713	tgfbr3	transforming growth factor beta receptor 3	X1
NC_031982.2	13468625	+	-0.957378066	exon (rna52026, exon 10 of 39)	6011	rna52026	LOC100703940	myomegalin	
NC_031981.2	15797912	+	-0.988646408	intron (rna49057, intron 3 of 5)	20411	rna49058	myf5	myogenic factor 5	
NC_031971.2	18854806	+	-1.005599902	Intergenic	-7210	rna13383	egr1-b	early growth response protein 1-B	

NC_031972.2	9051911	-	-1.012230543	exon (rna16352, exon 13 of 13)	18853	rna16355	LOC100706088	myocyte-specific enhancer factor 2C	
NC_031969.2	19957512	-	-1.064400875	intron (rna6969, intron 4 of 6)	12014	rna6971	pdgfa	platelet derived growth factor subunit A	X3
NC_031965.2	5641876	-	-1.075844488	intron (rna346, intron 3 of 17)	8535	rna346	megf10	multiple epidermal growth factor-like domains protein 10	
NC_031972.2	23475365	+	-1.098434563	intron (rna17631, intron 2 of 3)	7365	rna17631	fgf5	fibroblast growth factor 5	
NC_031969.2	19957365	-	-1.107855591	intron (rna6969, intron 4 of 6)	12161	rna6971	pdgfa	platelet derived growth factor subunit A	X3
NC_031981.2	15816458	+	-1.12446071	exon (rna49058, exon 3 of 3)	1865	rna49058	myf5	myogenic factor 5	
NC_031987.2	23463685	-	-1.134838183	intron (rna46988, intron 3 of 4)	27415	rna46988	fgf14	fibroblast growth factor 14	X2
NC_031973.2	16325252	-	-1.134960514	exon (rna22658, exon 6 of 6)	2900	rna22658	LOC100709026	myosin regulatory light chain 2 skeletal muscle isoform	
NC_031973.2	16363668	-	-1.175796197	intron (rna22670, intron 1 of 10)	3791	rna22670	tgfb1i1	transforming growth factor beta 1 induced transcript 1	X2
NC_031983.2	11645848	-	-1.178026983	exon (rna55197, exon 14 of 14)	-3052	rna55199	gfgrl1	fibroblast growth factor receptor-like 1	
NC_031986.2	40926094	+	-1.183218918	intron (rna66611, intron 1 of 2)	11942	rna66611	fgf22	fibroblast growth factor 22	
NC_031970.2	25446920	-	-1.19696553	intron (rna10853, intron 16 of 18)	-15514	rna10856	mdfic	myoD family inhibitor domain-containing protein	
NC_031976.2	14188066	+	-1.209240328	exon (rna30019, exon 2 of 2)	9093	rna30019	gdf6a	growth/differentiation factor 6-A	
NC_031975.2	32876563	-	-1.218291117	exon (rna28854, exon 2 of 2)	1405	rna28854	egr1	early growth response protein 1	
NC_031983.2	10433785	-	-1.23658196	intron (rna55019, intron 28 of 30)	26479	rna55013	tgfb3	transforming growth factor beta 3	
NC_031981.2	24627362	-	-1.256090733	exon (rna49806, exon 17 of 43)	7741	rna49813	pde4dip	myomegalin	
NC_031972.2	3616120	-	-1.272555825	Intergenic	-15601	rna15913	myod1	myogenic differentiation 1	X1
NC_031970.2	9129637	-	-1.328931107	intron (rna9196, intron 1 of 2)	-3027	rna9200	ngf	nerve growth factor	
NC_031969.2	30860219	-	-1.336462331	promoter-TSS (rna8101)	-379	rna8100	gas7	growth arrest-specific protein 7	
NC_031971.2	18856853	-	-1.342973603	Intergenic	-5163	rna13383	egr1-b	early growth response protein 1-B	
NC_031969.2	19963624	+	-1.346668877	intron (rna6970, intron 3 of 6)	5902	rna6971	pdgfa	platelet derived growth factor subunit A	X3
NC_031969.2	30860217	+	-1.352992955	promoter-TSS (rna8101)	-377	rna8100	gas7	growth arrest-specific protein 7	
NC_031987.2	14364738	-	-1.353997698	intron (rna46013, intron 2 of 15)	13481	rna46018	igf2bp2	insulin like growth factor 2 mRNA binding protein 2	X7
NC_031976.2	14168362	+	-1.361676942	Intergenic	28797	rna30019	gdf6a	growth/differentiation factor 6-A	

NC_031965.2	5670247	+	-1.385107684	Intergenic	-19836	rna346	megf10	multiple epidermal growth factor-like domains protein 10	
NC_031976.2	30160664	+	-1.389819707	intron (rna31688, intron 4 of 11)	36243	rna31693	mef2d	myocyte enhancer factor 2D	X2
NC_031970.2	25449265	-	-1.394986794	exon (rna10853, exon 19 of 19)	-13169	rna10856	mdfic	myoD family inhibitor domain-containing protein	
NC_031976.2	14168530	-	-1.397867342	Intergenic	28629	rna30019	gdf6a	growth/differentiation factor 6-A	
NC_031975.2	32876561	+	-1.398997208	exon (rna28854, exon 2 of 2)	1407	rna28854	egr1	early growth response protein 1	
NC_031972.2	23475426	-	-1.426065825	intron (rna17631, intron 2 of 3)	7426	rna17631	fgf5	fibroblast growth factor 5	
NC_031970.2	25413435	+	-1.462151797	intron (rna10853, intron 3 of 18)	-48999	rna10856	mdfic	myoD family inhibitor domain-containing protein	
NC_031972.2	2095659	-	-1.463155726	exon (rna15781, exon 7 of 7)	8947	rna15781	frs2	fibroblast growth factor receptor substrate 2	
NC_031986.2	40926138	-	-1.480326511	intron (rna66611, intron 1 of 2)	11986	rna66611	fgf22	fibroblast growth factor 22	
NC_031971.2	18854895	-	-1.485034152	Intergenic	-7121	rna13383	egr1-b	early growth response protein 1-B	
NC_031972.2	37984755	-	-1.5266982	intron (rna18665, intron 7 of 11)	9965	rna18667	LOC100708380	fibroblast growth factor receptor 1-A	
NC_031986.2	22143049	-	-1.53307153	exon (rna64962, exon 5 of 6)	2333	rna64962	LOC100698429	myosin light chain 3 skeletal muscle isoform	
NC_031965.2	5658444	+	-1.536048392	Intergenic	-8033	rna346	megf10	multiple epidermal growth factor-like domains protein 10	
NC_031983.2	7131489	+	-1.54128383	exon (rna54720, exon 7 of 18)	45172	rna54730	fgfr3	fibroblast growth factor receptor 3	X11
NC_031977.2	15942963	-	-1.548903043	intron (rna33674, intron 1 of 9)	8050	rna33675	mef2c	myocyte enhancer factor 2C	X20
NC_031970.2	25449263	+	-1.563525904	exon (rna10853, exon 19 of 19)	-13171	rna10856	mdfic	myoD family inhibitor domain-containing protein	
NC_031972.2	2097062	+	-1.584978092	TTS (rna15781)	10350	rna15781	frs2	fibroblast growth factor receptor substrate 2	
NC_031972.2	63178204	-	-1.592570045	exon (rna20965, exon 4 of 7)	-11322	rna20963	fgf6	fibroblast growth factor 6	X1
NC_031978.2	31443441	+	-1.597430041	Intergenic	-13597	rna38285	fgf8	fibroblast growth factor 8	
NC_031976.2	17590833	-	-1.612272717	intron (rna30285, intron 32 of 35)	10264	rna30287	igflr1	IGF like family receptor 1	
NC_031970.2	3028159	-	-1.633740187	Intergenic	21305	rna8728	gdf5	growth differentiation factor 5	
NC_031981.2	7714379	+	-1.67510828	intron (rna48438, intron 7 of 17)	-26790	rna48439	mdfic	myoD family inhibitor domain-containing protein	
NC_031969.2	19957363	+	-1.684335548	intron (rna6969, intron 4 of 6)	12163	rna6971	pdgfa	platelet derived growth factor subunit A	X3

NC_031965.2	5670333	-	-1.694711966	Intergenic	-19922	rna346	megf10	multiple epidermal growth factor-like domains protein 10	
NC_031981.2	24557801	+	-1.719977568	Intergenic	-6626	rna49804	pde4dip	myomegalin	
NC_031966.2	26301033	-	-1.720091227	Intergenic	9681	rna4403	egr1	early growth response 1	
NC_031981.2	7788420	-	-1.740474381	intron (rna48441, intron 7 of 7)	19925	rna48446	mdfic	myoD family inhibitor domain-containing protein	
NC_031972.2	2097311	-	-1.911577462	exon (rna15782, exon 33 of 34)	10599	rna15781	frs2	fibroblast growth factor receptor substrate 2	
NC_031973.2	22060953	+	-1.916722342	Intergenic	-41039	rna23332	gdf10	growth differentiation factor 10	
NC_031987.2	14364572	+	-1.994210817	intron (rna46013, intron 2 of 15)	13647	rna46018	igf2bp2	insulin like growth factor 2 mRNA binding protein 2	X7
NC_031972.2	63178202	+	-2.023477863	exon (rna20965, exon 4 of 7)	-11320	rna20963	fgf6	fibroblast growth factor 6	X1
NC_031972.2	37972756	-	-2.032014547	exon (rna18662, exon 12 of 17)	11008	rna18664	LOC100707841	fibroblast growth factor receptor 1-A	
NC_031987.2	25810038	-	-2.036029225	exon (rna47161, exon 17 of 23)	-6949	rna47159	gdf3	growth differentiation factor 3	
NC_031975.2	33815623	-	-2.054951942	intron (rna28982, intron 16 of 26)	25575	rna28982	LOC100709470	platelet-derived growth factor receptor beta	
NC_031987.2	25810106	-	-2.059284899	exon (rna47161, exon 17 of 23)	-7017	rna47159	gdf3	growth differentiation factor 3	
NC_031975.2	12064355	-	-2.074138036	promoter-TSS (rna27244)	-672	rna27244	ltbp3	latent transforming growth factor beta binding protein 3	X3
NC_031980.2	36388748	+	-2.075251886	Intergenic	-13858	rna44708	negr1	neuronal growth regulator 1	X1
NC_031987.2	25810046	+	-2.093633246	exon (rna47161, exon 17 of 23)	-6957	rna47159	gdf3	growth differentiation factor 3	
NC_031983.2	19853639	+	-2.117705556	exon (rna55899, exon 28 of 28)	12791	rna55897	ghsr	growth hormone secretagogue receptor type 1	
NC_031970.2	32144070	-	-2.127048445	intron (rna11561, intron 1 of 2)	-8684	rna11560	mdfic2	MyoD family inhibitor domain containing 2	
NC_031972.2	23472458	-	-2.152541584	exon (rna17631, exon 2 of 4)	4458	rna17631	fgf5	fibroblast growth factor 5	
NC_031973.2	22061114	-	-2.185916765	Intergenic	-40878	rna23332	gdf10	growth differentiation factor 10	
NC_031980.2	9160858	+	-2.202156216	Intergenic	-20793	rna42581	LOC100710008	transforming growth factor beta-3	
NC_031966.2	26300851	+	-2.206810975	Intergenic	9499	rna4403	egr1	early growth response 1	
NC_031972.2	23504377	-	-2.313878004	Intergenic	36377	rna17631	fgf5	fibroblast growth factor 5	
NC_031965.2	5671027	+	-2.31496574	Intergenic	-20616	rna346	megf10	multiple epidermal growth factor-like domains protein 10	
NC_031983.2	11645684	+	-2.320621812	exon (rna55197, exon 14 of 14)	-3216	rna55199	gfgrl1	fibroblast growth factor receptor-like 1	

NC_031969.2	23536548	+	-2.422669743	Intergenic	-19149	rna7377	myh	myosin heavy chain fast skeletal muscle	
NC_031972.2	23472343	+	-2.522619255	exon (rna17631, exon 2 of 4)	4343	rna17631	fgf5	fibroblast growth factor 5	
NC_031978.2	33449458	+	-2.524417493	intron (rna38363, intron 17 of 18)	-27172	rna38382	ltbp1	latent transforming growth factor beta binding protein 1	X1
NC_031970.2	9129489	+	-2.568009462	intron (rna9196, intron 1 of 2)	-2879	rna9200	ngf	nerve growth factor	
NC_031969.2	23538240	+	-2.719505282	Intergenic	19650	rna7378	myh	myosin heavy chain fast skeletal muscle	
NC_031979.2	37675829	+	-2.732381475	intron (rna41516, intron 1 of 4)	-6758	rna41517	fgf12	fibroblast growth factor 12	X2
NC_031985.2	17765661	-	-2.819671749	Intergenic	-5196	rna61813	LOC100690941	myocyte-specific enhancer factor 2D homolog	
NC_031980.2	36388750	-	-3.030957271	Intergenic	-13860	rna44708	negr1	neuronal growth regulator 1	X1
NC_031981.2	34689624	+	-3.193363613	exon (rna50487, exon 8 of 10)	11605	rna50488	gas2l3	growth arrest specific 2 like 3	X2
NC_031972.2	23518407	-	-3.23990151	Intergenic	50407	rna17631	fgf5	fibroblast growth factor 5	
NC_031985.2	12043478	-	-3.357086349	exon (rna61269, exon 56 of 77)	-11691	rna61265	gdf6	growth differentiation factor 6	
NC_031978.2	1189046	-	-3.42824603	Intergenic	-4752	rna35786	egr2b	early growth response protein 2b	
NC_031972.2	23518221	+	-3.714351321	Intergenic	50221	rna17631	fgf5	fibroblast growth factor 5	
NC_031984.2	21365373	-	-3.740497793	TTS (rna59101)	105	rna59100	LOC100709242	epidermal growth factor receptor kinase substrate 8-like protein 3	
NC_031972.2	2095585	-	-3.81240876	exon (rna15781, exon 7 of 7)	8873	rna15781	frs2	fibroblast growth factor receptor substrate 2	
NC_031969.2	23536834	-	-4.19797001	Intergenic	-19435	rna7377	myh	myosin heavy chain fast skeletal muscle	

7

Table S9. B)

Chromosome	Position	Strand	logFC	Annotated feature	Distance to TSS	Transcript	Gene symbol	Description	Transcript variant
NC_031981.2	7714510	+	1.174147565	intron (rna48438, intron 7 of 17)	-26659	rna48439	LOC100706760	myoD family inhibitor domain-containing protein	X2
NC_031965.2	16394571	+	1.096134499	TTS (rna1045)	7162	rna1048	grtp1	growth hormone regulated TBC protein 1	X3
NC_031987.2	6704894	-	1.01914623	Intergenic	-7549	rna45409	mstn	myostatin	
NC_031978.2	24703665	-	1.013825174	Intergenic	40293	rna37841	fgfr2	fibroblast growth factor receptor 2	X2
NC_031977.2	23412387	-	1.004418292	Intergenic	6945	rna34377	gadd45g	growth arrest and DNA damage inducible gamma	
NC_031978.2	925479	+	0.877284391	intron (rna35759, intron 1 of 2)	1715	rna35759	fgfbp3	fibroblast growth factor binding protein 3	
NC_031987.2	6704712	+	0.870802818	Intergenic	-7367	rna45409	mstn	myostatin	
NC_031981.2	7710282	+	0.780459791	exon (rna48438, exon 5 of 18)	-30887	rna48439	LOC100706760	myoD family inhibitor domain-containing protein	X2
NC_031969.2	19957365	-	-0.993846601	intron (rna6971, intron 4 of 5)	12161	rna6971	pdgfa	platelet derived growth factor subunit A	X3
NC_031972.2	23472458	-	-1.395081946	exon (rna17631, exon 2 of 4)	4458	rna17631	LOC100700362	fibroblast growth factor 5	
NC_031972.2	23504377	-	-1.832311266	Intergenic	36377	rna17631	LOC100700362	fibroblast growth factor 5	
NC_031979.2	37675829	+	-2.018102155	intron (rna41516, intron 1 of 4)	-6758	rna41517	fgf12	fibroblast growth factor 12	X2
NC_031972.2	2095585	-	-2.033650896	exon (rna15781, exon 7 of 7)	8873	rna15781	frs2	fibroblast growth factor receptor substrate 2	
NC_031978.2	1189046	-	-2.132177366	Intergenic	-4752	rna35786	LOC100712147	early growth response protein 2b	

Table S9. C)

Chromosome	Position	Strand	logFC	Annotated feature	Distance to TSS	transcript	Gene symbol	Description	Transcript variant
NC_031984.2	21365373	-	2.786768591	TTS (rna59101)	105	rna59100	LOC100709242	epidermal growth factor receptor kinase substrate 8-like protein 3	
NC_031981.2	15816458	+	2.152285451	exon (rna49058, exon 3 of 3)	1865	rna49058	myf5	myogenic factor 5	
NC_031970.2	9129489	+	1.908390935	intron (rna9196, intron 1 of 2)	-2879	rna9200	LOC100696124	nerve growth factor	X4
NC_031976.2	30125013	-	1.818987486	intron (rna31693, intron 1 of 12)	592	rna31693	mef2d	myocyte enhancer factor 2D	X2
NC_031972.2	54257903	-	1.545323561	intron (rna20146, intron 2 of 20)	12892	rna20146	LOC100711993	insulin-like growth factor 1 receptor	
NC_031982.2	13468625	+	1.541320301	exon (rna52023, exon 10 of 40)	6011	rna52026	LOC100703940	myomegalin	X8
NC_031982.2	13468712	-	1.464095837	exon (rna52023, exon 10 of 40)	6098	rna52026	LOC100703940	myomegalin	X8
NC_031987.2	14364738	-	1.384543393	intron (rna46016, intron 2 of 15)	13481	rna46019	igf2bp2	insulin like growth factor 2 mRNA binding protein 2	X6
NC_031987.2	6704894	-	1.290658284	Intergenic	-7549	rna45409	mstn	myostatin	
NC_031977.2	18676629	+	1.216397161	intron (rna33954, intron 1 of 4)	10203	rna33954	gas2l1	growth arrest specific 2 like 1	
NC_031977.2	15942963	-	1.144007371	intron (rna33661, intron 1 of 9)	8050	rna33675	mef2c	myocyte enhancer factor 2C	X20
NC_031978.2	24703665	-	1.038002001	Intergenic	40293	rna37840	fgfr2	fibroblast growth factor receptor 2	X1
NC_031985.2	17740430	-	1.003569426	intron (rna61810, intron 7 of 11)	20034	rna61814	LOC100690941	myocyte-specific enhancer factor 2D homolog	X5
NC_031981.2	7710282	+	0.907078077	exon (rna48438, exon 5 of 18)	-30887	rna48439	LOC100706760	myoD family inhibitor domain-containing protein	X2
NC_031966.2	2128899	-	-1.421372511	Intergenic	9373	rna2695	LOC100707945	fibroblast growth factor 4-like	
NC_031976.2	17588156	-	-1.47287885	exon (rna30285, exon 29 of 36)	12941	rna30287	igflr1	IGF like family receptor 1	
NC_031978.2	33510485	-	-3.40653547	intron (rna38384, intron 3 of 29)	33853	rna38385	ltbp1	latent transforming growth factor beta binding protein 1	X5
NC_031965.2	5670962	-	-4.346180809	Intergenic	-20551	rna346	LOC100701872	multiple epidermal growth factor-like domains protein 10	

Table S10. List of muscle samples (n=5) and their RNA-Seq library characterization in docx format. The table provides information for the precise number of reads and fragments during every step of analysis, from raw sequenced to uniquely aligned reads and assigned fragments.

Sample	Raw Reads	Trimmed Reads	Trimming (%)	Unique Mapped	Multiple Mapped	PE one mate mapped	Overall Alignment rate (%)	Total fragments	Assigned fragments	Assigned fragments (%)
M1	20873309	20184106	96.70	11233133	1684865	347123	65.93	24730025	11064020	44.7
M2	49791573	47903749	96.21	38096988	6167339	1653372	95.39	63993176	37544846	58.7
M3	19577511	18742472	95.73	14962010	2444564	443955	95.17	24928917	14640811	58.7
M4	18209404	17532554	96.28	14758124	1739326	492615	95.5	21169447	14440598	68.2
M5	21686206	21017566	96.92	17043449	2196846	707358	94.65	26636391	16569450	62.2

Table S11. List of the top 30 most expressed transcripts in fast muscle (n=5) in docx format.

The table provides information regarding the expression of each transcript based on normalized counts as well as their annotation within the Nile tilapia genome.

Transcripts	GeneRef	Gene Symbol	Description	Transcript Variant
rna1710	gene843	LOC100534413	actin alpha skeletal muscle A	
rna7931	gene4056	mylpf	myosin light chain phosphorylatable fast skeletal muscle	
rna7376	gene3774	LOC100712344	myosin heavy chain fast skeletal muscle	
rna41226	gene20698	LOC100702938	creatine kinase M-type	
rna7378	gene3776	LOC100707599	myosin heavy chain fast skeletal muscle	
rna22679	gene11375	LOC100706607	sarcoplasmic/endoplasmic reticulum calcium ATPase 1	X2
rna22680	gene11375	LOC100706607	sarcoplasmic/endoplasmic reticulum calcium ATPase 1	X1
rna26546	gene13383	LOC100701380	creatine kinase M-type	
rna64962	gene32753	LOC100698429	myosin light chain 3 skeletal muscle isoform	
rna15907	gene8033	LOC100707421	troponin T fast skeletal muscle isoforms	X1
rna15902	gene8033	LOC100707421	troponin T fast skeletal muscle isoforms	X2
rna15908	gene8033	LOC100707421	troponin T fast skeletal muscle isoforms	X5
rna15909	gene8033	LOC100707421	troponin T fast skeletal muscle isoforms	X3
rna15903	gene8033	LOC100707421	troponin T fast skeletal muscle isoforms	X4
rna15910	gene8033	LOC100707421	troponin T fast skeletal muscle isoforms	X6
rna15911	gene8033	LOC100707421	troponin T fast skeletal muscle isoforms	X8
rna15905	gene8033	LOC100707421	troponin T fast skeletal muscle isoforms	X7
rna15912	gene8033	LOC100707421	troponin T fast skeletal muscle isoforms	X10
rna15906	gene8033	LOC100707421	troponin T fast skeletal muscle isoforms	X9
rna15904	gene8033	LOC100707421	troponin T fast skeletal muscle isoforms	X11
rna20017	gene10051	tpm1	tropomyosin 1	X7
rna20015	gene10051	tpm1	tropomyosin 1	X4
rna8448	gene4318	LOC100697135	nucleoside diphosphate kinase B	
rna20020	gene10051	tpm1	tropomyosin 1	X1
rna20018	gene10051	tpm1	tropomyosin 1	X5
rna20016	gene10051	tpm1	tropomyosin 1	X2
rna31010	gene15698	gapdh	glyceraldehyde-3-phosphate dehydrogenase	
rna20023	gene10051	tpm1	tropomyosin 1	X9
rna22736	gene11408	ald	fructose-bisphosphate aldolase A	
rna20019	gene10051	tpm1	tropomyosin 1	X3

Supplementary figures

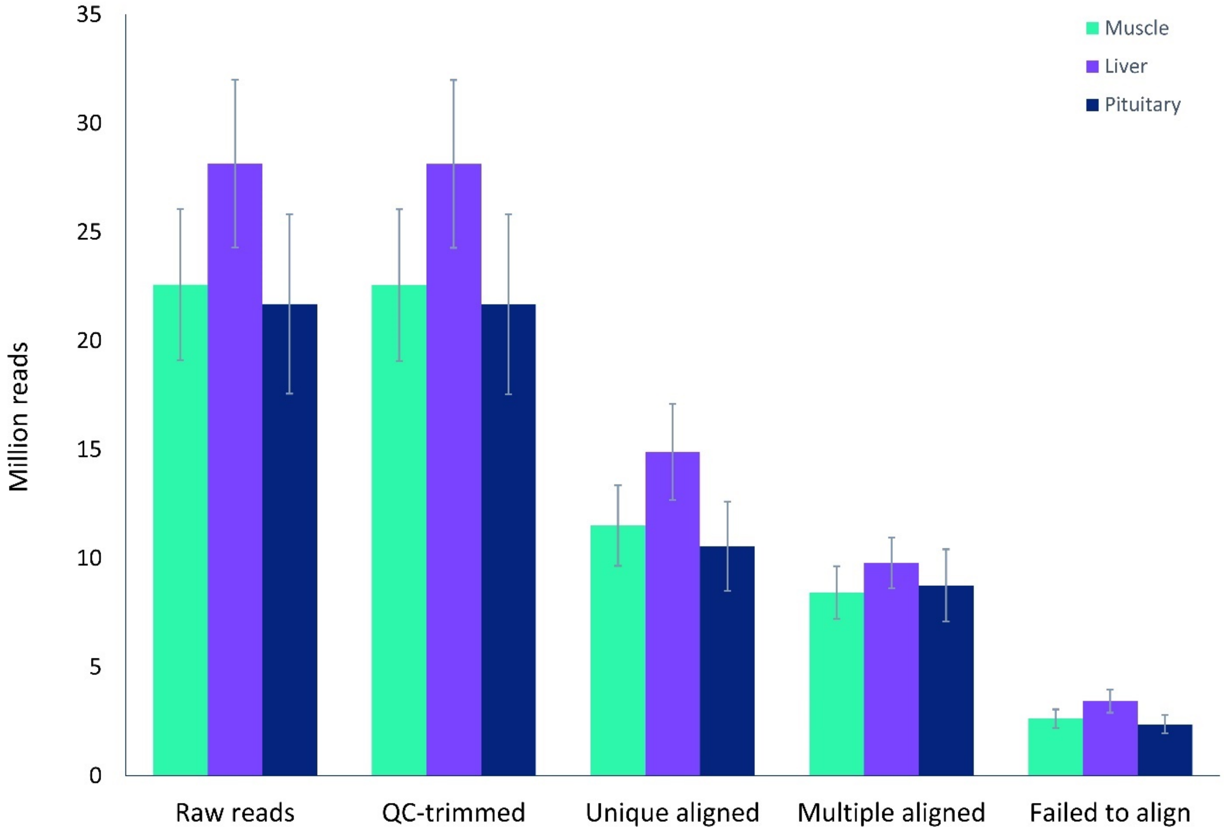

Figure S1. Barplot representing the RRHP dataset. Raw, quality control and trimmed, uniquely aligned, multiple aligned and failed to align reads are shown on the x-axis while the number of reads in millions is shown on the y-axis across the three tissues. Muscle, liver and pituitary are represented in green, purple and dark blue, respectively (n=5). Bars and error bars represent means and standard errors, respectively.

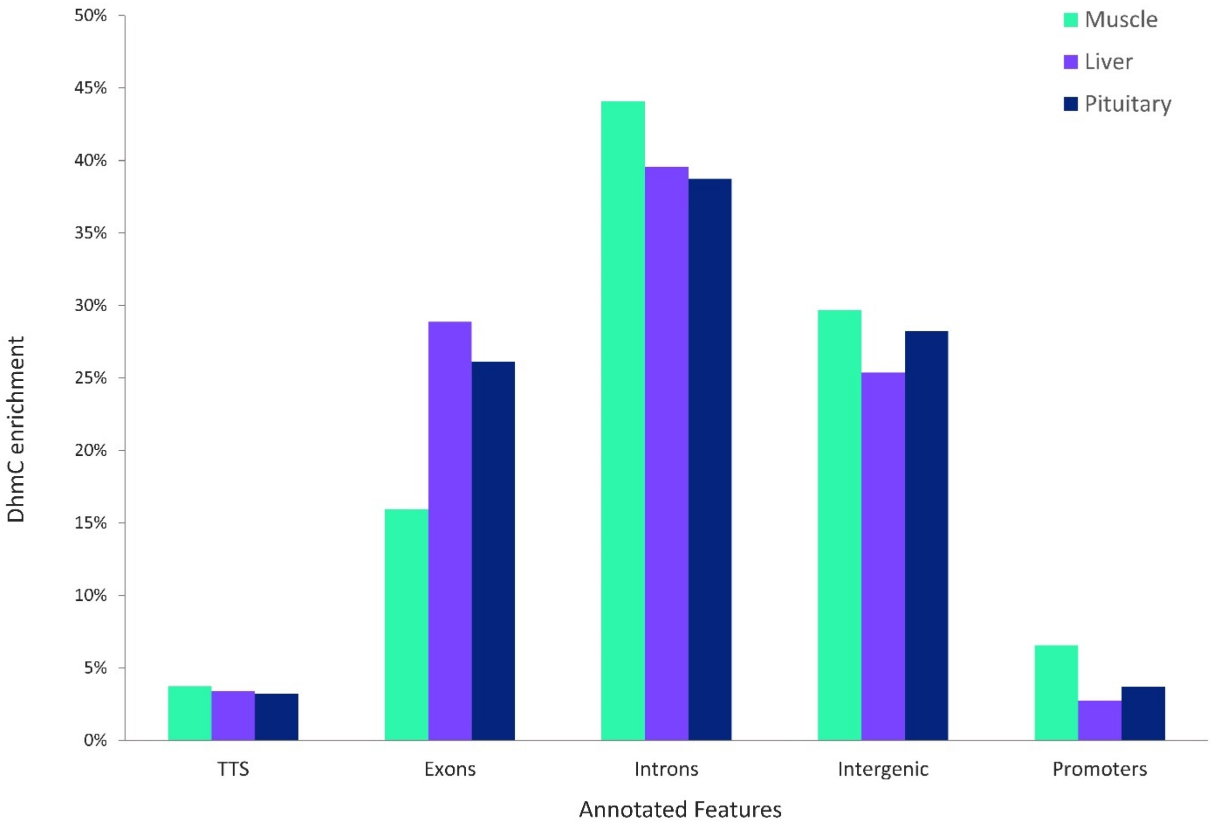

Figure S2. Barplot representing the percentage of DhmC enrichment (y-axis) within the annotated features (TTS, exons, introns, intergenic, promoters; x-axis). Muscle, liver and pituitary are represented in green, purple and dark blue, respectively (n=5).
